# Phenome-wide association study of population-differentiating genetic variants around gene *ACSL1*

**DOI:** 10.1093/emph/eoae024

**Published:** 2024-09-20

**Authors:** Shuang Yang, Houjian Cai, Kaixiong Ye

**Affiliations:** Department of Genetics, Franklin College of Arts and Sciences, University of Georgia, Athens, GA 30602, USA; Department of Pharmaceutical and Biomedical Sciences, College of Pharmacy, University of Georgia, Athens, GA 30602, USA; Department of Genetics, Franklin College of Arts and Sciences, University of Georgia, Athens, GA 30602, USA; Institute of Bioinformatics, University of Georgia, Athens, GA 30602, USA

**Keywords:** positive selection, phenome-wide association study, *ACSL1*, diabetes, menopause

## Abstract

**Background and objectives:**

Demographic dynamics and natural selection during human evolution shaped the present-day patterns of genetic variations, and geographically varying genetic factors contribute to different disease prevalences across human populations. This study aims to evaluate the presence of positive selection on the gene encoding long-chain fatty acyl-CoA synthetase 1 (*ACSL1*) and the phenotypic impacts of population-differentiating genetic variants around this gene.

**Methodology:**

Three types of statistical tests for positive selection, based on site frequency spectrum, extended haplotype homozygosity and population differentiation, were applied to the whole-genome sequencing data from the 1000 Genomes Project. A phenome-wide association study of *ACSL1* was performed with published genome-wide association studies (GWAS) and transcriptome-wide association studies, including phenome-wide studies in biobanks.

**Results:**

Genetic variants associated with *ACSL1* expression in various tissues exhibit geographically varying allele frequencies. Three types of statistical tests consistently supported the presence of positive selection on the coding and regulatory regions of *ACSL1* in African, European, South Asian and East Asian populations. A phenome-wide association study of *ACSL1* revealed associations with type 2 diabetes, blood glucose, age at menopause, mean platelet volume and mean reticulocyte volume. The top allele associated with lower diabetes risk has the highest frequency in European populations, whereas the top allele associated with later menopause has the highest frequency in African populations.

**Conclusions and implications:**

Positive selection on *ACSL1* resulted in geographically varying genetic variants, which may contribute to differential phenotypes across human populations, including type 2 diabetes and age at menopause.

## INTRODUCTION

Demographic dynamics and natural selection during human evolution shaped the present-day patterns of genetic variations, and geographically varying genetic factors contribute to different disease prevalence across human populations [1]. Natural selection of adaptive alleles in a historically stable local environment drove up the allele frequencies and forged gene–environment matching relationships that maintained population fitness. However, the rapid changes in living environment and lifestyle in modern human societies create gene–environment mismatches that may partly explain the current epidemics of chronic diseases [2, 3]. Large allele frequency changes could also happen as a result of random genetic drift, especially in the context of founder events, population bottlenecks or admixture [1]. The accumulation of whole-genome sequencing data from modern and ancient DNA samples in global human populations has enabled the identification of genetic variants with geographically varying frequency differences and the characterization of the shaping evolutionary events [4, 5]. On the other hand, genome-wide association studies (GWAS), which scan across the genome to identify genetic variants associated with a trait or disease of interest, have surged in number over the last two decades. The open science practice of sharing genome-wide summary statistics after a GWAS is published has significantly boosted reproducibility and downstream analysis [6]. These summary statistics typically include the effect size estimate, standard error or confidence internal and the *P* value for each variant included in a GWAS. The availability of GWAS of many traits, especially those in biobank-scale cohorts with extensive phenotypes, enables the extraction of all published phenotypic associations for genetic variants of interest and thus offers an unprecedented opportunity to systemically interrogate the phenotypic impacts of population-differentiating genetic variants [7, 8].

Genes involved in fatty acid metabolism, such as fatty acid desaturases (FADS), have been shown to be frequent targets of positive selection during human evolution [9–13]. Long-chain fatty acyl-CoA synthetases, encoded by *ACSL1*, *ACSL3*, *ACSL4*, *ACSL5* and *ACSL6*, is a family of rate-limiting enzymes in fatty acid metabolism that convert long-chain (i.e. 10–20 carbons) fatty acids to their corresponding fatty acyl-CoAs. Our previous study showed that *ACSL1* has the broadest spectrum of fatty acid substrates and that its expression level is elevated in prostate tumors. We further demonstrated that the knockdown of *ACSL1* inhibited prostate cancer cell proliferation and the growth of xenograft tumors by suppressing the biosynthesis of various acyl-CoAs, decreasing intracellular lipid accumulation and reducing β-oxidation [14]. In addition to prostate cancer, *ACSL1* has been implicated in the pathogenesis of various cancers, including lung, breast, ovary and liver [15–18]. The phenotypic impacts of *ACSL1* beyond cancer are much less studied. To date, there is no phenome-wide association study of genetic variants around *ACSL1*, which would yield profound insights into the clinical relevance of *ACSL1*. In addition, while we were investigating the genetic regulation of *ACSL1* expression, we noticed that SNPs associated with *ACSL1* expression exhibit drastic allele frequency differences across human populations. We hypothesize that *ACSL1* was subject to positive natural selection during human evolution.

In this study, by leveraging whole-genome sequencing data of 2504 individuals from the 1000 Genomes Project [19], we formally evaluated the presence of positive selection on *ACSL1* in four continent-level human populations, including African, European, South Asian and East Asian populations. Three types of statistical tests were performed, which are complementary and detect different signatures of positive selection [4, 20]. The first type of test is based on the site frequency spectrum (SFS) and searches for an excess of rare variants or high-frequency derived alleles. It includes Tajima’s *D* [21] and Fay and Wu’s *H* [22]. The second type of test searches for extended haplotype homozygosity and includes integrated haplotype score (iHS) [23] and number of segregating sites by length (nSL) [24]. The third is a population differentiation-based test, called the population branch statistic (PBS) [25], which identifies genetic variants with extreme allele frequency changes in one population in comparison to two control populations. In addition to evolutionary analysis, we performed a phenome-wide association study of genetic variants around *ACSL1* by leveraging published GWAS and transcriptome-wide association studies (TWAS) of thousands of phenotypes, including phenome-wide association studies in biobank-scale cohorts. We systematically extracted phenotypic associations of *ACSL1* genetic variants from these existing GWAS and TWAS. Our findings revealed the presence of positive selection on *ACSL1* in all four human populations, and that population-differentiating genetic variants around *ACSL1* are associated with diabetes, blood glucose, menopause and blood-cell sizes.

## METHODOLOGY

### Expression and genetic regulation of *ACSL1* across tissues

The adult Genotype-Tissue Expression (GTEx) project characterized transcriptome and genetic regulation of gene expression in 54 non-diseased tissue sites with about 1000 adult individuals [26]. The expression levels and expression quantitative trait loci (eQTL) data utilized in our study were retrieved from the GTExPortal (version 8). The eQTLGen Consortium performed eQTL analyses in up to 31 684 blood and peripheral blood mononuclear cell samples from 37 cohorts [27]. Summary statistics (i.e. effect size estimates and *P* values) utilized in our study were retrieved from the phase I database.

### Statistical tests for positive selection

Three types of statistical tests for positive selection were applied to sequencing data from the 1000 Genomes Project, which includes 2504 individuals representing 5 continental regions and 26 global populations [19]. Three SFS-based statistics, including nucleotide diversity (*π*), Tajima’s *D* [21] and Fay and Wu’s *H* [22], were calculated using a sliding-window approach with a window size of 5 Kb and a moving step of 1 Kb. Haplotype-based tests, including the iHS [23] and nSL [24], were calculated using the software selscan version 1.1.0a [28]. Only common SNPs with minor allele frequency >5% were included in the calculation. Genetic variants without ancestral allele information were excluded from analyses. The population differentiation-based test, the PBS [25], was calculated for the African population (*N* = 661), European (*N* = 503) and East Asian populations (*N* = 504) using pairwise *F*_ST_. The PBS for the South Asian population (*N* = 489) was calculated in comparison to the African and European populations. Statistical significance for these statistics was evaluated by comparing an observed value to the genome-wide distribution, and an empirical *P* value was derived as the proportion of genome-wide variants or regions with more extreme values.

### Phenotypic associations of genetic variants around *ACSL1*

The NHGRI-EBI GWAS Catalog curates published GWAS, and it had collected 6899 publications, 621 136 top associations and 86 887 full summary statistics as of 7 June 2024 [29]. Of note, summary statistics refer to the effect size estimate, its corresponding standard error and *P* value for each variant analyzed in a GWAS. GWAS ATLAS is another database of publicly available GWAS summary statistics. It contains 4756 GWAS from 473 unique studies across 3302 unique traits and 28 domains in its latest release (Release 3) on 15 November 2019 [30]. Open Targets Genetics is an integrative resource that aggregates GWAS and functional omics data to prioritize causal variants and genes for GWAS loci. At the time of this study, the Version 8 covers 57 244 GWAS, of which 8894 have full or fine-mapping summary statistics. These GWAS were compiled from three sources, including GWAS Catalog, UK Biobank and FinnGen [31, 32]. GWAS from the UK Biobank encompassed 2139 binary case-control phenotypes and 1283 quantitative traits [33, 34]. The R6 data freeze of FinnGen had GWAS of over 2800 disease phenotypes [35]. Phenotypic associations of *ACSL1* were retrieved from these three databases by searching the gene name on 13 June 2024. The three databases are complementary, maintained by different research groups or organizations with their own curating processes, and updated at different schedules. Entries in each database have references to the original GWAS. While some associations from the same GWAS are reported in two or three databases, others may be curated only in one database. We treated these associations equally regardless of the number of databases reporting them.

Depending on availability, genome-wide GWAS summary statistics of selected traits were downloaded, and the associations around *ACSL1* were visualized with LocusZoom [36]. Specifically, summary statistics were downloaded from the NHGRI-EBI GWAS Catalog on 14 June 2024, for study GCST90018958 of hemoglobin A1c levels (HbA1c) [37], study GCST006867 of type 2 diabetes (T2D) [38], study GCST90002346 of mean platelet volume [39] and study GCST90002396 of mean reticulocyte volume [40]. For age at menopause (last menstrual period), summary statistics were downloaded from the MRC IEU OpenGWAS for study ukb-b-17422 [41, 42].

A TWAS integrates GWAS and eQTLs to identify associations between a phenotype and the predicted expression level of a gene in a specific tissue. TWAS hub evaluated 342 traits and expression levels of genome-wide genes in 74 healthy or cancer tissues or cell lines [43]. The PhenomeXcan platform synthesized GWAS of 4091 traits with transcriptome regulation data of 49 tissues from GTEx (v8) [44]. Phenotypes associated with the predicted expression of *ACSL1* in various tissue contexts were retrieved on 17 June 2024. Data visualizations were prepared using R (version 4.2.0) unless stated otherwise.

## RESULTS

### eQTLs of *ACSL1* exhibit large frequency differences across human populations

**Figure 1.**
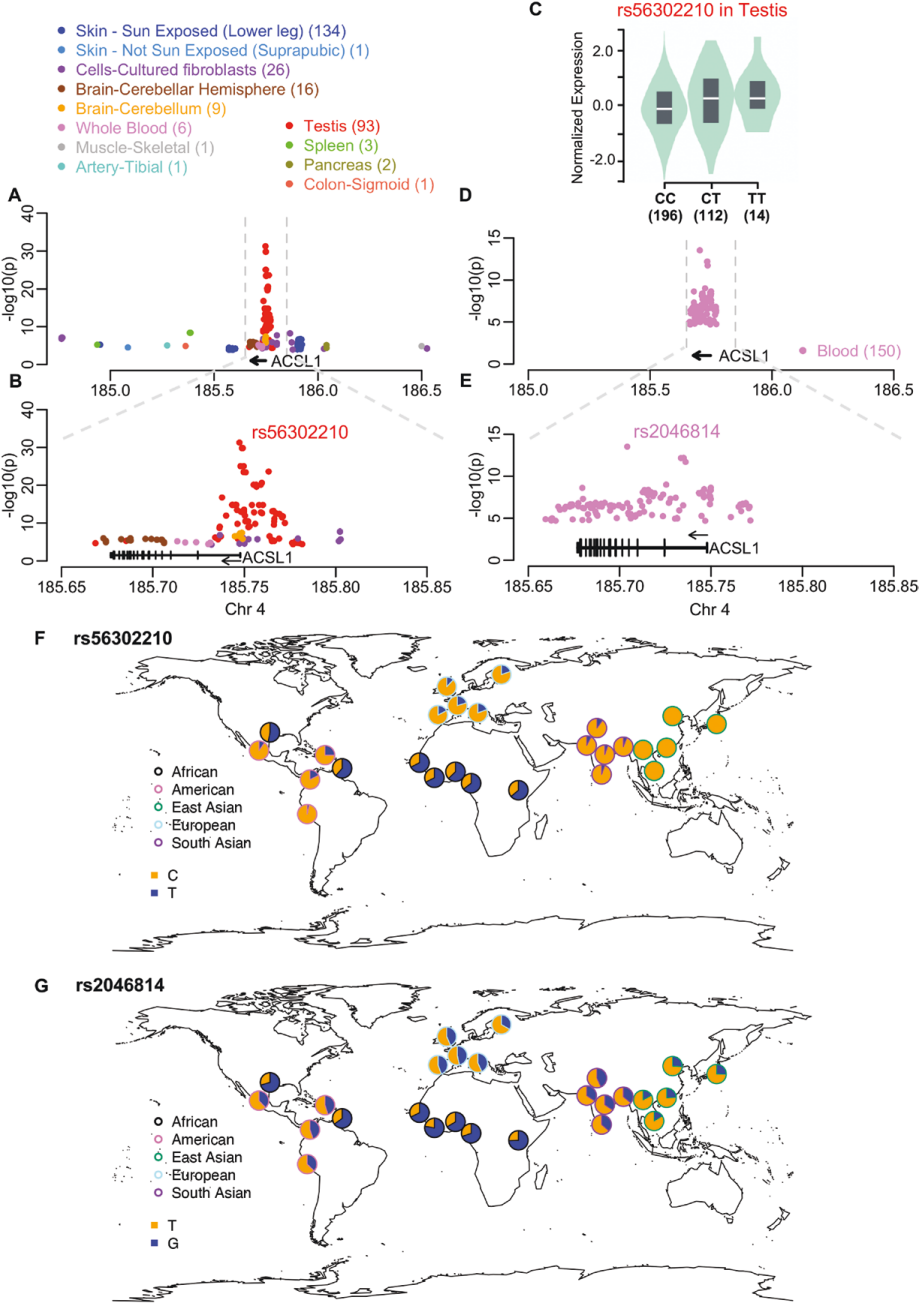
Genetic variants associated with the expression of *ACSL1* in various tissues and cell lines. (A) All associated variants as detected in the GTEx project. Colors indicate the tissues or cells. The number of associated variants in each tissue or cell line is indicated in the corresponding parenthesis. None of the variants has significant associations in more than one tissues or cells. (B) A zoom-in look at the 200-kb region surrounding the *ACSL1* gene. The model of the longest transcript is shown at the bottom. (C) The genotype-expression association of the most significant SNP, rs56302210. The number of individuals in each genotype group is indicated at the bottom. (D) All variants associated with expression of *ACSL1* in the blood as detected in the eQTLGen study. (E) A zoom-in look at the eQTL signals. (F) The global frequency distribution for the most significant variant in GTEx, rs56302210. (G) The global frequency distribution for the most significant variant in eQTLGen, rs2046814

The expression and regulation of *ACSL1* in a wide range of human tissues and cells were investigated using data from the GTEx project [26]. *ACSL1* is ubiquitously expressed in all 54 tissues and cells examined. Whole blood, liver, adipose tissues and skeletal muscle have the highest expression levels, whereas most brain tissues have the lowest (Supplementary Figure S1). Analysis of eQTLs identified genetic variants associated with the expression level of *ACSL1* in 12 tissues and cells. The strongest eQTL signals are in testis and the peak SNP is rs56302210 (*C*/*T*; *P* = 5.2e-32) (Fig. 1A and B, and Supplementary Fig. S2). The T allele of rs56302210 is associated with higher *ACSL1* expression in the testis (Fig. 1C). Signals of eQTLs in testis cluster around the transcription start site of *ACSL1*, overlapping active regulatory elements and transcription factor binding sites. Notably, the peak SNP is located in a binding site for *MYC*, a proto-oncogene (Supplementary Fig. S3). In the eQTLGen study that performed eQTL analysis in over 31 000 blood samples, a cluster of eQTLs was identified for *ACSL1* (Fig. 1D, E). The peak SNP is rs2046814 (*G*/*T*; *P* = 2.95e-14), and the T allele is associated with higher *ACSL1* expression in the blood. Interestingly, we noticed that these two SNPs have drastically different frequencies across global populations. For rs56302210, the expression-increasing allele in the testis (T) has the highest frequency in Africans (63.54%), has a much lower frequency in Europeans (17.89%), and is totally absent in East Asians (0%) (Fig. 1F). For rs2046814, the expression-increasing allele in the blood (*T*) has the highest frequency in East Asians (78.1%) and the lowest frequency in Africans (29.8%). Similar patterns of drastically different allele frequencies across populations were observed for other top eQTLs (Supplementary Fig. S4). In summary, genetic variants associated with the expression of *ACSL1* exhibit large frequency differences across human populations.

### Positive selection on *ACSL1* in four continental human populations

**Figure 2.**
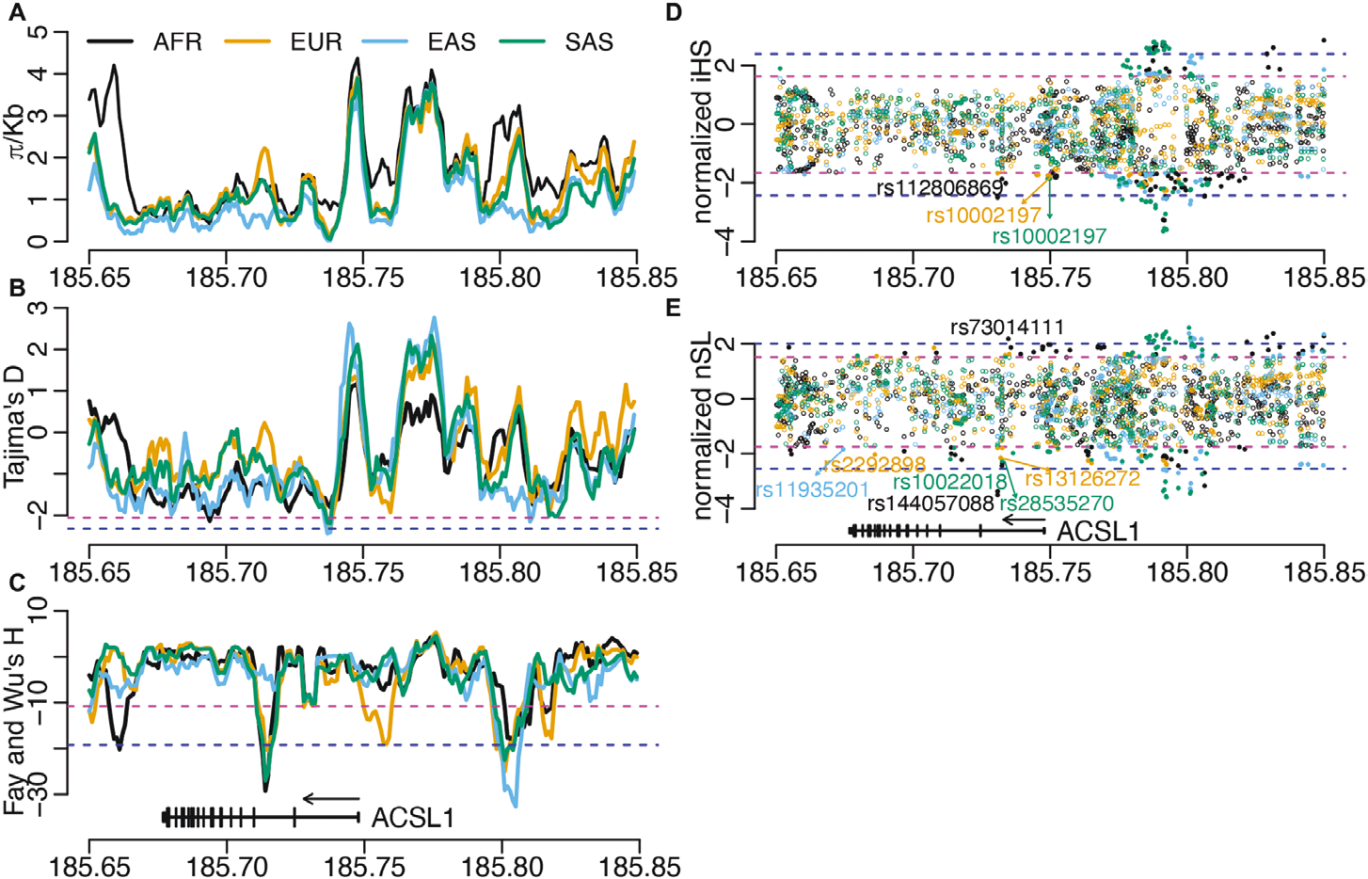
Signals of positive selection surrounding *ACSL1* in four human populations. (A) Nucleotide diversity. (B) Tajima’s D. (C) Fay and Wu’s H. (D) iHS. (E) nSL. Results for four human populations, African (AFR), European (EUR), East Asian (EAS) and South Asian (SAS) are shown in different colors. The magenta dashed lines with less extreme values indicate the cutoffs for the top (or bottom) 5% of the genome-wide distribution of the corresponding statistics. The blue dashed lines with more extreme values indicate the top (or bottom) 1%. The distributions and cutoffs are population-specific, and the most stringent cutoffs among the four populations are used in the plots so that any values beyond the shown cutoffs are statistically significant. Some representative SNPs are labeled in (D) and (E) to help in orientation

The possible presence of positive selection on *ACSL1* in four continental populations, including African, European, East Asian and South Asian populations, was systematically evaluated with whole-genome sequencing samples from the 1000 Genomes Project. We performed three types of statistical tests for positive selection, respectively based on SFS, haplotype homozygosity and population differentiation. Based on the SFS, there is a slight decrease in nucleotide diversity (*π*) in the gene body of *ACSL1* (Fig. 2A). An SFS-based test, Tajima’s *D*, revealed two clusters of extremely negative values in the gene body. The one around Chr4:185.694 Mb was observed in African and East Asian populations, whereas the other one around Chr4:185.737 Mb was observed in all populations except the African population (Fig. 2B). Another SFS-based test, Fay and Wu’s *H*, unraveled a cluster of extreme negative values in the second intron in all populations except the East Asian population. In addition, there is a cluster of extreme values right around the transcription start site, and this signal of positive selection is unique to the European population (Fig. 2C). A haplotype-based test, iHS, showed one cluster of extreme values in intron 1 around SNP rs112806869, and another cluster at the transcription start site around SNP rs10002197 (Fig. 2D). The other haplotype-based test, nSL, demonstrated multiple clusters of extreme values across the gene body (Fig. 2E). These positive selection signals detected by haplotype-based tests are mainly present in African, European and South Asian populations. Of note, beyond the gene body and the transcription start site, signals of positive selection were also observed 50 Kb upstream of *ACSL1* in all four populations.

**Figure 3.**
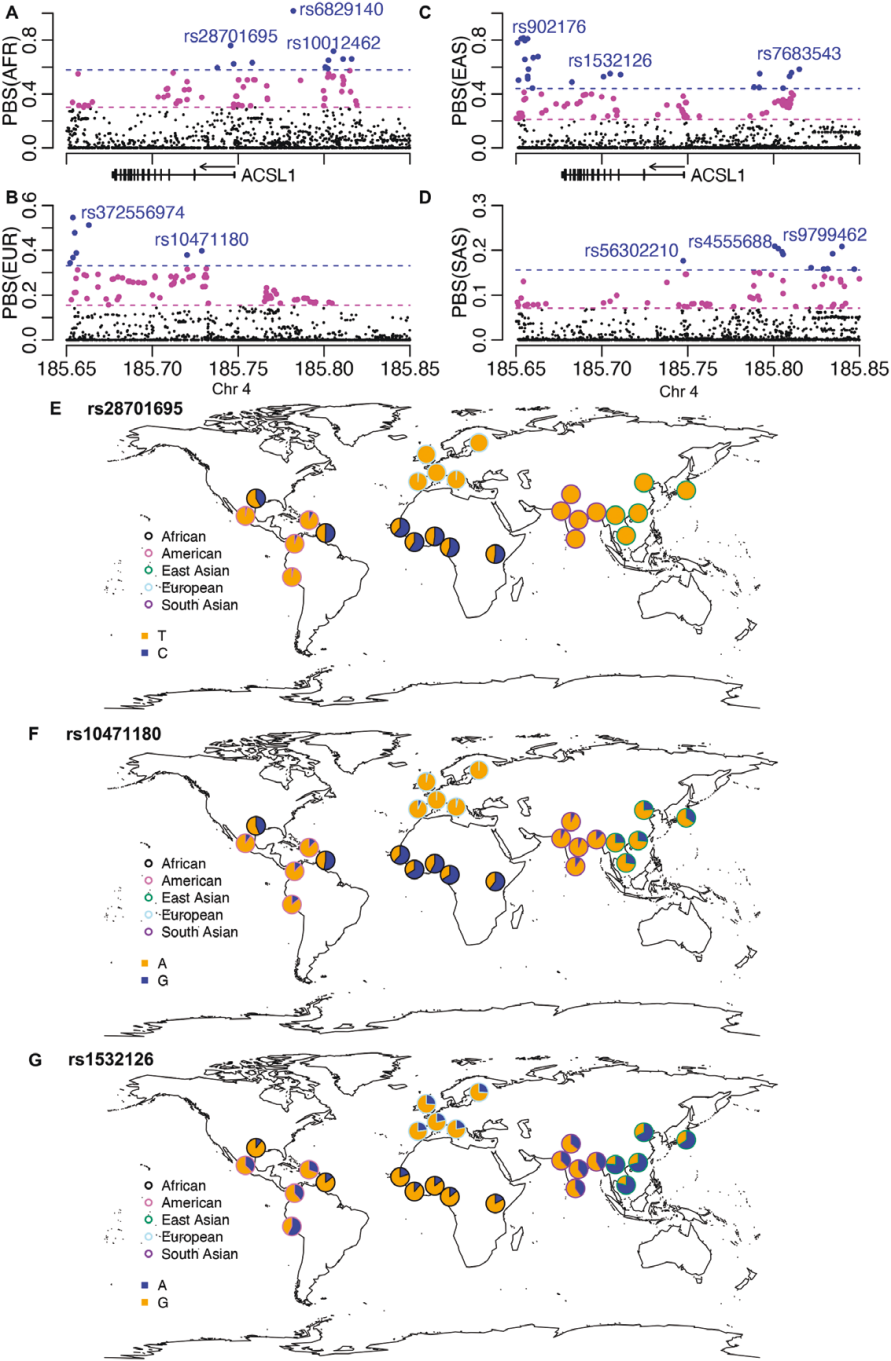
Genetic variants with extreme frequency changes in four human populations. PBS, a population differentiation-based test for extreme frequency changes, is shown for (A) African, (B) European, (C) East Asian and (D) South Asian populations. The magenta dashed lines with less extreme values indicate the cutoffs for the top 5% in the genome-wide distribution, while the blue dashed lines with more extreme values indicate the top 1%. Variants passing these cutoffs are indicated with either magenta or blue colors. The global frequency distributions are shown for top variants in *ACSL1*, including (E) rs28701695 in African, (F) rs10471180 in European and (G) rs1532126 in East Asian populations. The top variant in the South Asian population has been shown in Figure 1F

We applied the PBS, a population differentiation-based method, to identify genetic variants that experienced extreme frequency change in each of the four continental populations. Genetic variants with genome-wide top 1% extreme frequency change were identified in the gene body of *ACSL1* in all four populations (Fig. 3A–D). The top variant in the African population, rs28701695, has a C allele frequency of 53.3% in the African population, but this allele is almost absent in other populations (Fig. 3E). For the top variant in the European population, rs10471180, its A allele almost reaches fixation in the European population (97.3%), while its frequency is 72.8% in East Asian and 41.7% in African populations (Fig. 3F). The top variant in the East Asian population, rs1532126, has the highest frequency of A allele (71.6%) in the corresponding population (Fig. 3G). The top variant in the South Asian population, rs56302210, is also the top eQTL for *ACSL1* in the testis (Fig. 1F). Notably, the same variant (rs56302210) also has extreme PBS values for African and East Asian populations, reflecting its drastic frequency difference across global populations. In summary, the three types of statistical tests consistently supported the presence of positive selection on the regulatory and coding regions of *ACSL1*. The PBS test further identified a list of genetic variants with extreme frequency differences across populations.

### Phenome-wide association study of *ACSL1*

**Figure 4.**
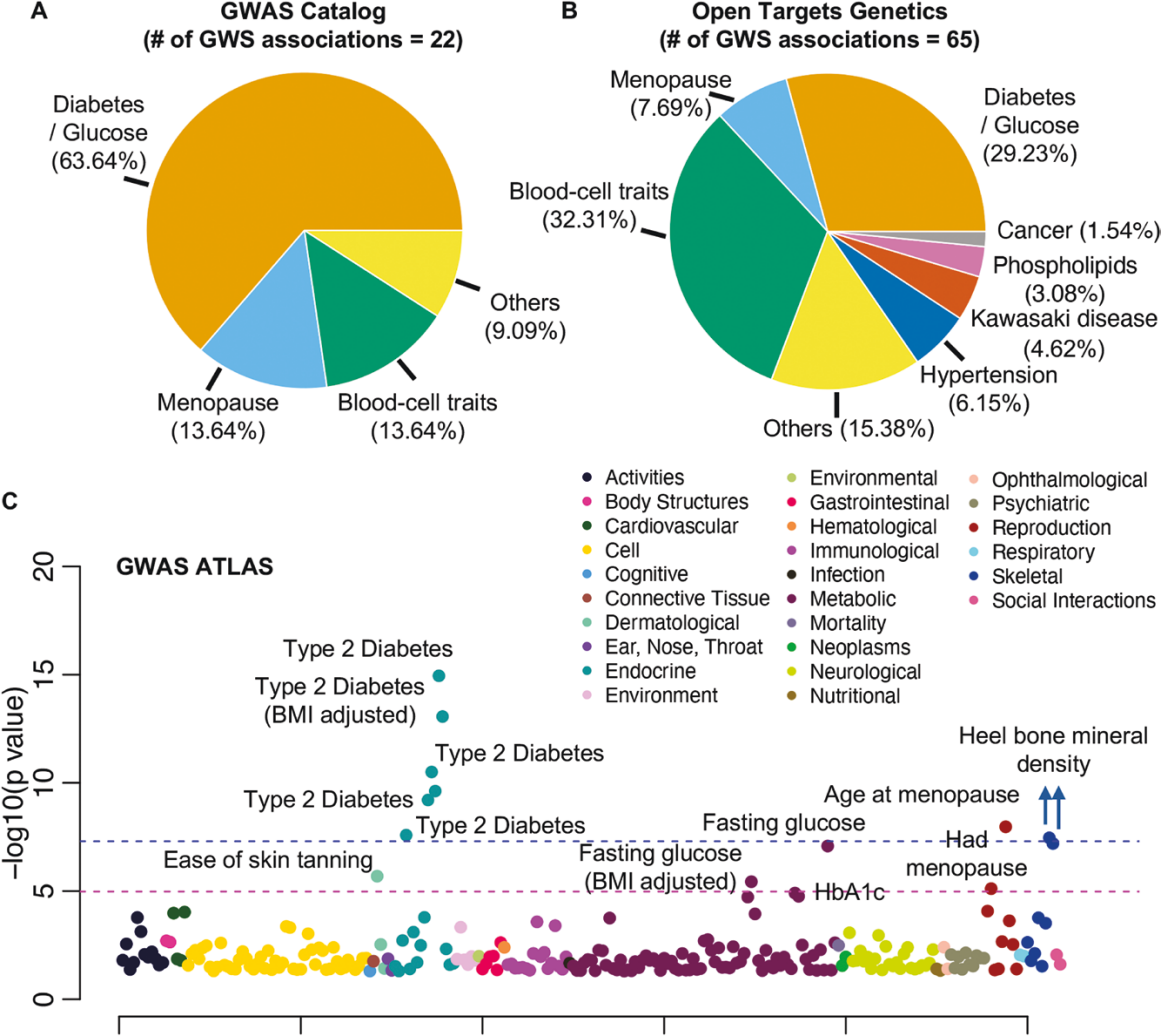
Phenotypes associated with genetic variants around *ACSL1*. Pie charts of genome-wide significant associations were shown for (A) GWAS Catalog and (B) Open Targets Genetics. (C) Manhattan plot was shown for all suggestive associations (*P* < 0.05) reported by GWAS ATLAS. Phenotypes were grouped by categories as indicated by the legend. The order within each category is arbitrary. GWS is indicated by the blue dashed line with a more extreme value. Bonferroni-corrected significance is indicated by the magenta dashed line with a less extremem value for the number of GWAS considered

To identify phenotypes associated with genetic variants around gene *ACSL1*, we queried three databases of published or publicly available GWAS summary statistics, including GWAS Catalog, GWAS ATLAS and Open Targets Genetics. These databases included phenome-wide GWAS of binary disease outcomes and quantitative traits in UK Biobank and FinnGen. The GWAS Catalog returned 24 associations for *ACSL1*, 22 of which reached genome-wide significance (GWS, *P* < 5.0e-8, Supplementary Table S1). Among these 22 GWS associations, 14 are related to diabetes or glucose, 3 are related to menopause and 3 are blood-cell traits (Fig. 4A). The two associations below GWS are related to type 1 diabetes (*P* = 4.0e-6 for SNP rs12644905) and neuroblastoma (*P* = 5.0e-6 for SNP rs7660927). In the Open Targets Genetics database, 65 GWS associations were reported (Supplementary Table S2). The commonly associated trait groups are blood-cell traits (*N* = 21), diabetes and glucose (*N* = 19) and menopause (*N* = 5) (Fig. 4B). There is one cancer-related association with chronic lymphocytic leukemia (*P* = 4.0e-8 for SNP rs57214277). Similarly, GWAS ATLAS returned the most significant associations with diabetes, glucose and menopause. It also reported associations with heel bone mineral density (Fig. 4C, Supplementary Table S3).

**Figure 5.**
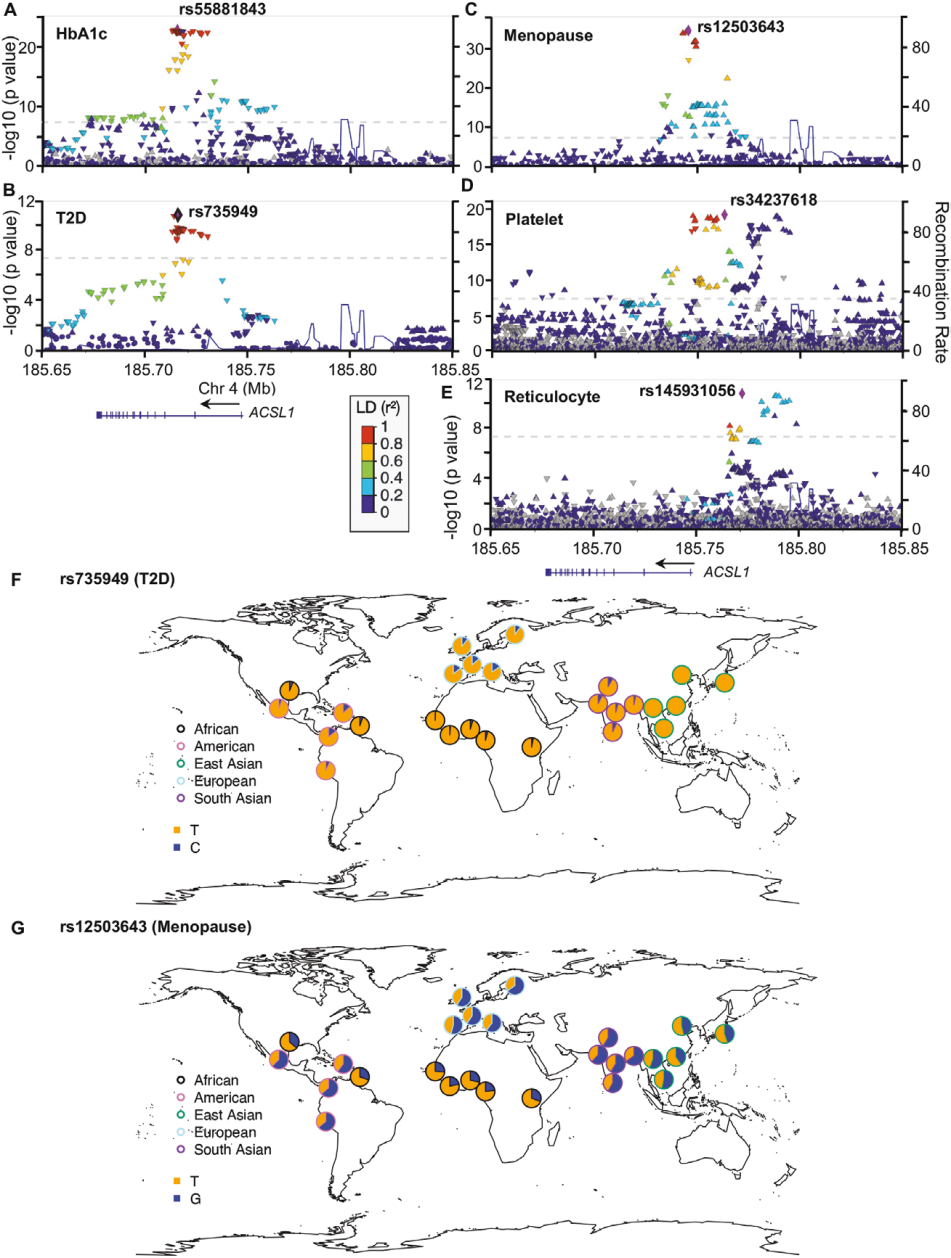
Association signals around *ACSL1*. LocusZoom plots are shown for (A) HbA1c, (B) T2D, (C) age at menopause, (D) mean platelet volume and (E) mean reticulocyte volume. The global frequency distributions are shown for the top variants of (F) T2D and (G) age at menopause

Phenotypic associations around *ACSL1* were visualized and examined for selected GWAS of HbA1c, T2D, age at menopause, mean platelet volume and mean reticulocyte volume (Fig. 5A–E). The association signals for HbA1c and T2D peak at the Intron 2 (Fig. 5A and B). The signals for the age at menopause are right at the transcription start site (Fig. 5C). The mean platelet volume has a wide association peak covering the region from Intron 1 to 50 kb upstream of *ACSL1* (Fig. 5D). The signals for mean reticulocyte volume are clustered around 20 Kb upstream of *ACSL1* (Fig. 5E). The top variant for HbA1c is rs55881843 (other allele/effect allele = *G*/*A*, *β* =  0.033, *P* = 1.49e-23), while the top variant for T2D is rs735949 (*T*/*C*, odds ratio = 0.93, *P* = 1.95e-11). These two SNPs are in perfect linkage disequilibrium (LD, *r*^2^ = 1) in the European population. The HbA1c-decreasing allele of rs55881843 (A) and the T2D-decreasing allele of rs735949 (C) has the highest frequency (12.9%) in the European population and much lower frequencies in other populations, 6.3%, 3.3% and 0% in South Asian, African and East Asian populations, respectively (Fig. 5F). The top variant for the age of menopause is rs12503643 (*G*/*T*, *β* = 0.047, *P* = 2.6e-35). The allele associated with later menopause (T) has the highest frequency in the African population (72.2%) and the lowest in European (40.3%) and South Asian (39.9%) populations (Fig. 5G). The top variant for mean platelet volume is rs34237618 (*T*/*C*, *β* =  0.023, *P* = 6.7e-24), and the one for mean reticulocyte volume is an insertion–deletion polymorphism rs145931056 (TCCTATGCCCTCC/T, *β* = 0.018, *P* = 1.9e-11). These two variants have similar geographical frequency distributions. The platelet-decreasing allele and the reticulocyte-increasing allele have frequencies of about 20% in the European population but are close to absent in African and East Asian populations.

In addition to direct associations between genetic variants and a phenotype, as evaluated by GWAS, the associations of multiple eQTLs could be aggregated to assess if the predicted expression of a gene in a specific tissue is associated with a phenotype. To examine phenotypes associated with the predicted expression of *ACSL1*, we queried two TWAS databases, including TWAS hub and PhenomeXcan. The top associations reported by TWAS hub include diabetes, HbA1c, a measure of heel bone mineral density (heel T-score), and height (Supplementary Fig. S5), while the top associations reported by PhenomeXcan are age at menopause, mean platelet volume and ‘had menopause’ (Supplementary Fig. S6). In summary, genetic variants around *ACSL1* are associated with diabetes, menopause and blood-cell traits, and the top associated variants exhibit geographically varying frequencies.

## DISCUSSION

Our study performed three types of statistical tests for positive selection and presented strong evidence for the presence of positive selection on the coding and regulatory regions of *ACSL1* in all four continental populations, including African, European, South Asian and East Asian populations. These events of genetic adaptation in local populations resulted in genetic variants with drastically different allele frequencies across human populations. Some of these variants are associated with the expression levels of *ACSL1* in various tissues, especially in the blood and testis. Phenome-wide association study further revealed that some of these population-differentiating genetic variants are associated with type 2 diabetes, blood glucose levels, age at menopause and the sizes of platelets and reticulocytes.

Across the three types of statistical tests for positive selection in four continental populations, there are multiple clusters of selection signals within a 200-Kb region surrounding *ACSL1*. Focusing on the gene body and the immediate upstream region of the transcription start site, SFS-based tests (i.e. Tajima’s *D* and Fay and Wu’s *H*) and haplotype-based tests (i.e. iHS and nSL) revealed positive selection signals in all four human populations. The signals in African, European and South Asian populations have strong support from all these four tests, whereas the signals in the East Asian population are mainly supported by Tajima’s *D*. However, the population differentiation-based test (i.e. PBS) identified variants with extreme allele frequency changes in all four populations, supporting the presence of positive selection in all of them. These signals are likely to be from separate natural selection events in each of the four human populations, instead of one selection event in the common ancestral population based on the following three reasons. First, the same genomic regions or genetic variants do not always have the same significant selection signals across populations. For example, Fay and Wu’s *H* unraveled a cluster of signals in Intron 2 in three other populations but not in East Asian. Second, a single selection event in the ancestral population would have reduced population differentiation in this genomic region. This is not the case, as shown by the population-differentiating variants identified by PBS. Third, current human populations shared a common ancestor in Africa at least 60 000 years ago, and the statistical tests utilized in this study could not detect such an ancient selection event [2, 4]. It is tempting to speculate that a common environmental factor was responsible for the separate selection events in four continental human populations. Due to the role of *ACSL1* in fatty acid metabolism and the well-established case of positive selection on fatty acid metabolic genes [9–13], it is possible that a dietary pattern as a result of the Agricultural Revolution exerted selection pressure on *ACSL1*. Future evolutionary and mechanistic studies are needed to examine the timing of the onset of positive selection, to pinpoint the causal adaptive variants, and to elucidate the beneficial traits. In addition, going beyond the gene body of *ACSL1*, selection signals are present at Chr4:185.80 Mb, 50 Kb upstream, and at Chr4:185.65 Mb, 25 Kb downstream of *ACSL1*. The possibility cannot be ruled out that these selection signals are related to *ACSL1* because eQTLs for *ACSL1* could be located over 1 Mb away (Fig. 1A). Identifying causal regulatory variants underlying the eQTL association signals will clarify if they and their target gene (i.e. *ACSL1*) are responsible for the positive selection signals.

Our phenome-wide association study of *ACSL1* identified genetic associations with type 2 diabetes, blood glucose, menopause and blood-cell traits. The top alleles associated with lower HbA1c and lower risk of T2D have the highest frequencies in European populations and much lower frequencies in African and East Asian populations. Interestingly, it is well-known that European populations have a lower prevalence of type 2 diabetes than Asian or African American populations [45]. Genetic associations of *ACSL1* variants with fasting glucose, diabetes and subclinical atherosclerosis were previously reported but without GWS [46]. Previous mice studies found that in mice fed with a high-fat diet, *ACSL1* expression is enhanced in white adipose tissue [47] and that *ACSL1* knockdown in adipocytes decreases insulin-stimulated cellular intake of glucose [47, 48]. The associations between *ACSL1* variants and menopause have not been reported before. We found that the allele associated with later menopause has the highest frequency in African populations and the lowest frequencies in both European and South Asian populations. However, previous epidemiologic studies did not find conclusive evidence supporting different ages of menopause across populations [49, 50]. Our study also, for the first time, identified associations of *ACSL1* variants with blood-cell sizes, including mean platelet volume and mean reticulocyte volume. Notably, there are two associations related to phospholipids, including 1-palmitoyl-2-linoleoyl-gpc (16:0/18:2) levels and Phosphatidylcholine-O_44:5_[M + H]1+/Phosphatidylcholine-P_44:4_[M + H]1 + levels. These associations are biologically convincing based on the role of ACSL1 in fatty acid metabolism. On the other hand, given the well-established role of *ACSL1* in various cancers [14–18], it is a bit surprising that only two associations are related to cancer, neuroblastoma at suggestive significance (*P* = 5.0e-6) and chronic lymphocytic leukemia at GWS. This may imply that *ACSL1* does not play a critical role in the initiation of tumorigenesis but, instead, in the maintenance of cancer cells. Our current phenome-wide association study only enumerated the traits associated with genetic variants around *ACSL1*, future mechanistic studies are required to characterize the causal variants, molecular mechanisms and pathophysiological pathways for each trait.

In our current results, we highlighted the top or most significant variants in each type of analysis. It is of interest to identify variants that have significant signals across gene expression regulation, positive selection and genotype-phenotype association. These variants may serve as candidates for follow-up mechanistic studies. For this purpose, we provided the list of eQTLs or trait-associated *ACSL1* genetic variants that also have evidence of positive selection in any of the four continental human populations (Supplementary Table S4). Some notable variants include rs145931056 (associated trait: mean reticulocyte volume), rs7665170 (platelet crit), rs4862423 (fasting glucose, HbA1c and type 2 diabetes) and rs7660927 (neuroblastoma). However, these candidate variants are only suggestive. This overlap analysis is complicated by a few limitations. First, different sets of genetic variants were included in different types of analysis. For example, different GWAS may use different genotyping platforms or imputation reference panels. Also, our haplotype-based selection tests (i.e. iHS and nSL) only included common variants with minor allele frequencies larger than 5%. Second, it is a common practice for GWAS databases (e.g. GWAS Catalog) to only report the top associated variant for each cluster of association signals. To formally evaluate the overlap of selection and association signals, co-localization analysis is required [51]. Moreover, statistical fine-mapping and integrative analysis with functional omics data will assist with the prioritization of candidate variants for experimental characterization [52].

Finally, there is a significant caveat regarding our genotype-phenotype association findings. Since existing GWAS and biobanks are overrepresented with European-ancestry participants, genetic associations in other ethnicities may not be available yet [8]. For example, the top differentiated variant in the African population, rs28701695, has a C allele frequency of 53.3% in the African but almost 0% in other populations. Its phenotypic associations could only be examined in the African population. Future genetic association studies in currently understudied ancestry groups will reveal more phenotypic impacts of population-differentiating genetic variants around *ACSL1*.

## CONCLUSIONS

Positive selection on *ACSL1* in four continental human populations during evolution has resulted in genetic variants with drastically different allele frequencies across populations. These population-differentiating genetic variants are associated with type 2 diabetes, blood glucose, menopause and blood-cell sizes. These variants may contribute to the different trait levels and disease prevalence across populations. Future studies are warranted to investigate the molecular mechanism of *ACSL1* and its genetic variants in diabetes, menopause and hemopoiesis.

## Supplementary Material

eoae024_suppl_Supplementary_Material

## Data Availability

All datasets used in this study are publicly available. Links are as follows: The Genotype-Tissue Expression (GTEx) Portal, https://gtexportal.org/home/; eQTLGen Phase 1, https://www.eqtlgen.org/phase1.html; 1000 Genomes Project, ftp://ftp.1000genomes.ebi.ac.uk/vol1/ftp/release/20130502/; Geography of Genetic Variants Browser, https://popgen.uchicago.edu/ggv/; NHGRI-EBI GWAS Catalog, https://www.ebi.ac.uk/gwas/; Open Targets Genetics, https://genetics.opentargets.org/; GWAS ATLAS, https://atlas.ctglab.nl/; IEU Open GWAS Project, https://gwas.mrcieu.ac.uk/; TWAS hub, http://twas-hub.org/; PhenomeXcan, http://apps.hakyimlab.org/phenomexcan/; Key scripts for data analysis and visualization are available publicly on GitHub, https://github.com/yekaixiong/ACSL1.
