# Supplementary material for: Phenome-wide association study of population-differentiating genetic variants around gene ACSL1
Source: Evol Med Public Health. 2024 Sep 20;12(1):178–90. doi: 10.1093/emph/eoae024 (PMC11462608; doi:10.1093/emph/eoae024)
Supplement: eoae024_suppl_Supplementary_Material [file eoae024_suppl_supplementary_material.docx]

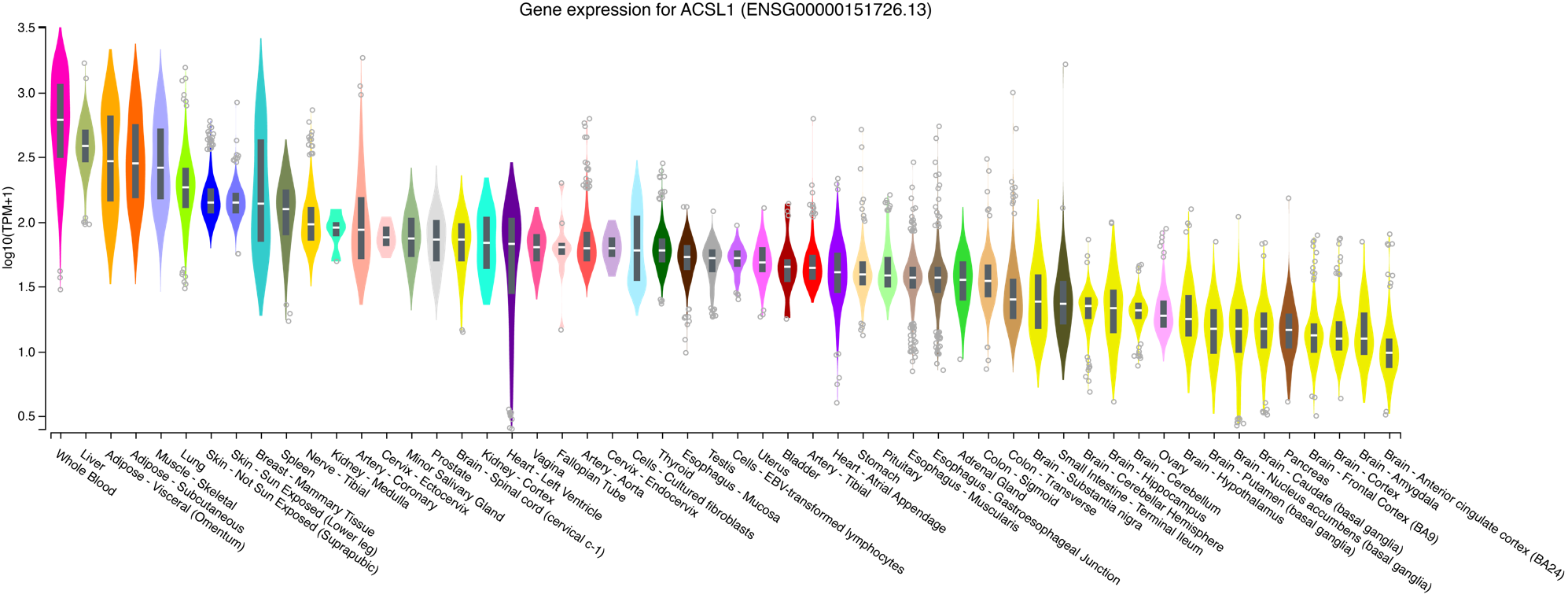


Supplementary Figure S1. The expression of *ACSL1* in 54 human tissues and cells. TPM: transcripts per million.


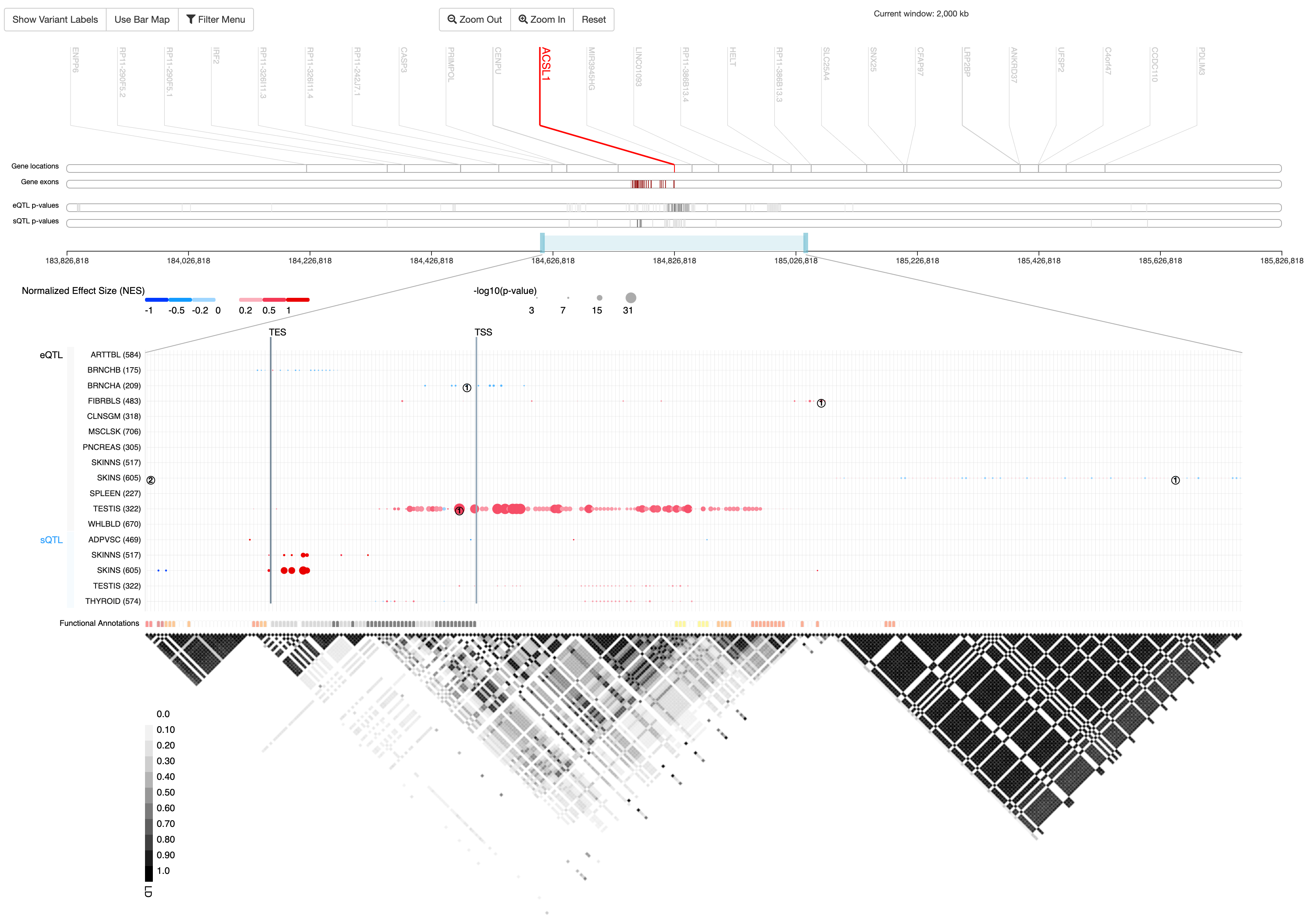


Supplementary Figure S2. Expression and splicing quantitative trait loci (eQTLs and sQTLs) for *ACSL1*. Only tissues and cells with statistically significant eQTLs or sQTLs are shown. Red indicates that the reference allele is associated with higher expression level while blue indicates the opposite. The size of the bubble corresponds to statistical significance. Please note that eQTLs in other tissues are not as significant as those in testis. Heat maps at the bottom indicate the linkage disequilibrium of genetic variants in this genomic region.


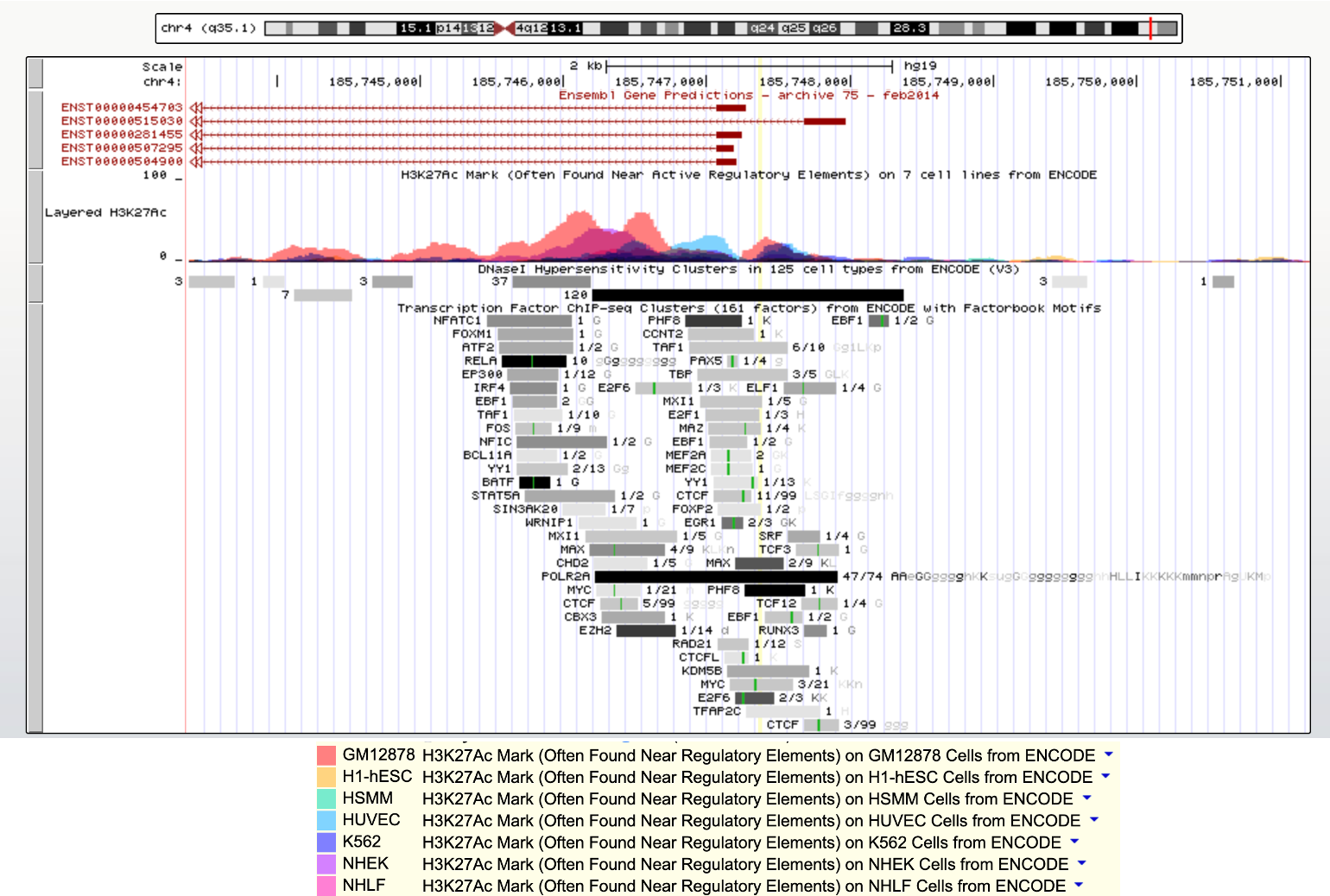


Supplementary Figure S3. Regulatory elements around the transcription start site of *ACSL1*. The figure shows a UCSC genome browser view of the *ACSL1* region. The two tracks shown are: 1) H3K27Ac Mark (Often Found Near Active Regulatory Elements) on 7 cell lines from ENCODE, and 2) Transcription Factor ChIP-seq Clusters (161 factors) from ENCODE with Factorbook Motifs. The position of the SNP rs56302210 is indicated with the yellow vertical line.


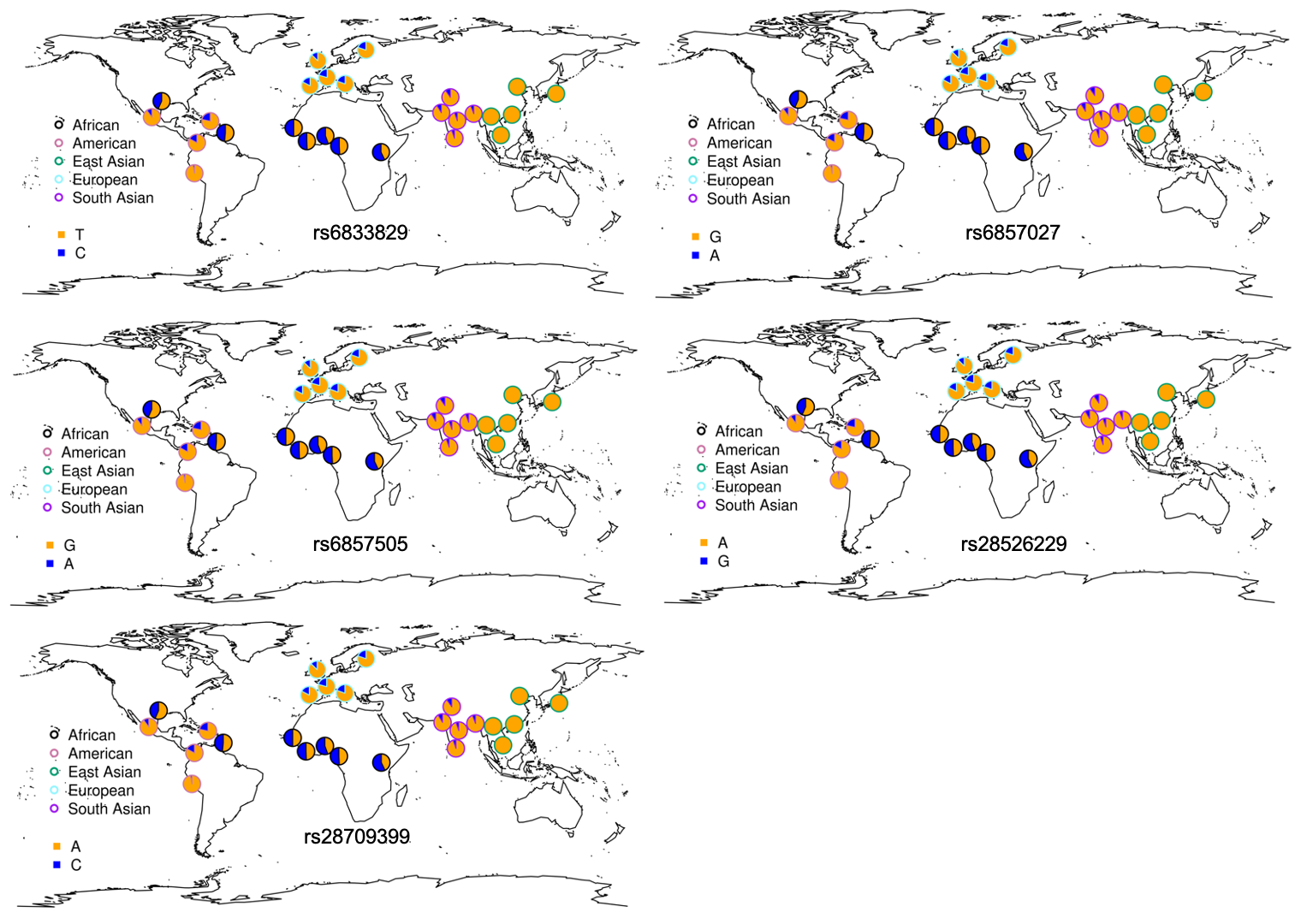


Supplementary Figure S4. Global frequency distribution for five eQTLs that share the second most significant p value in GTEx (*p* = 1.5e-30): rs6833829 (expression-increasing allele / expression-decreasing allele: C/T), rs6857027 (A/G), rs6857505 (A/G), rs28526229 (G/A), and rs28709399 (C/A)


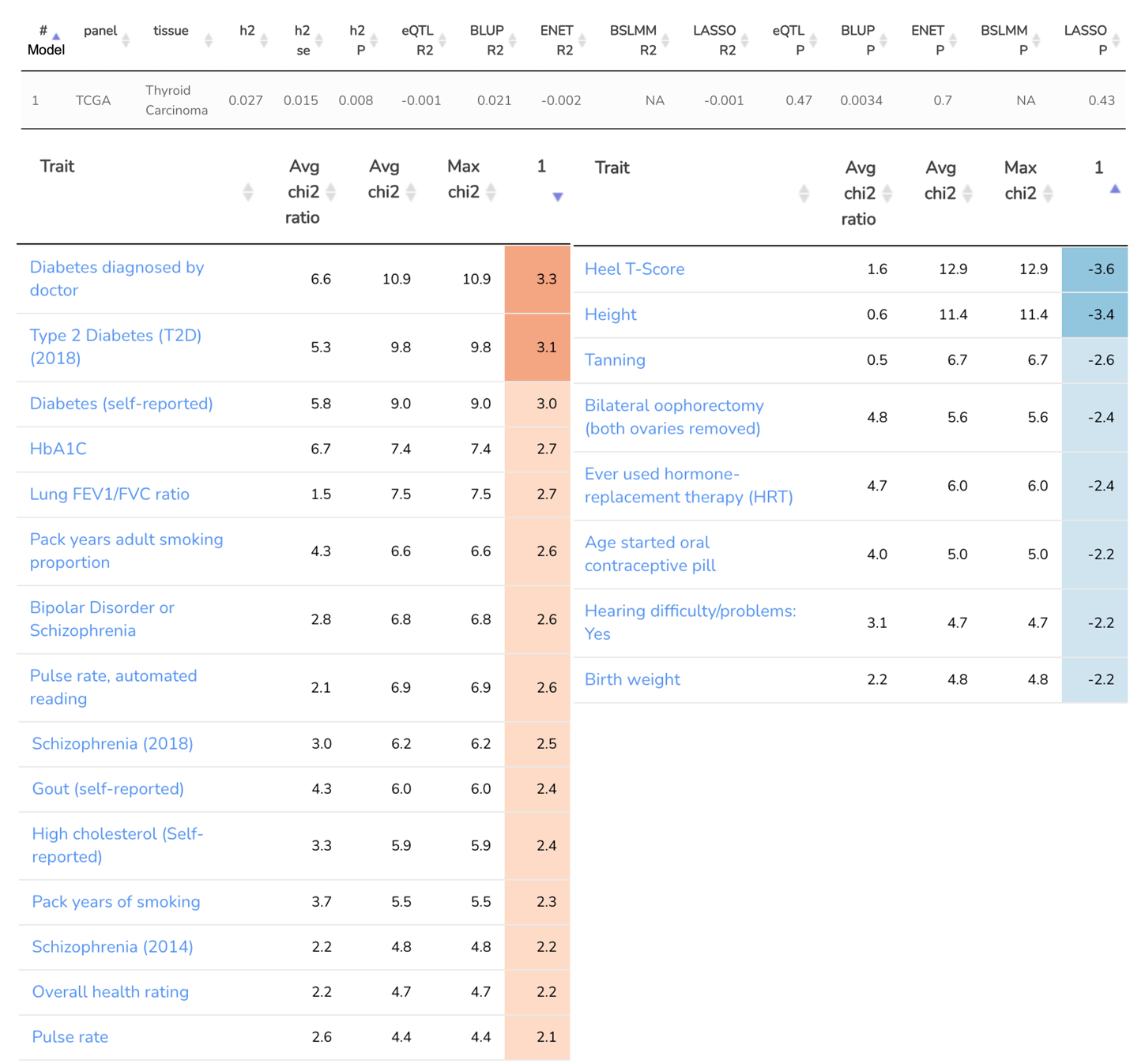


Supplementary Figure S5. Phenotypes associated with the predicted expression of *ACSL1*, as reported in TWAS hub. There is only one model (Model #1) that can predict the expression of *ACSL1*, and it is for the expression in thyroid carcinoma. The last column indicates the z score of association between the phenotype and the predicted expression, with red color indicating positive association and blue indicating negative association. Only phenotypes with significant associations are shown here.


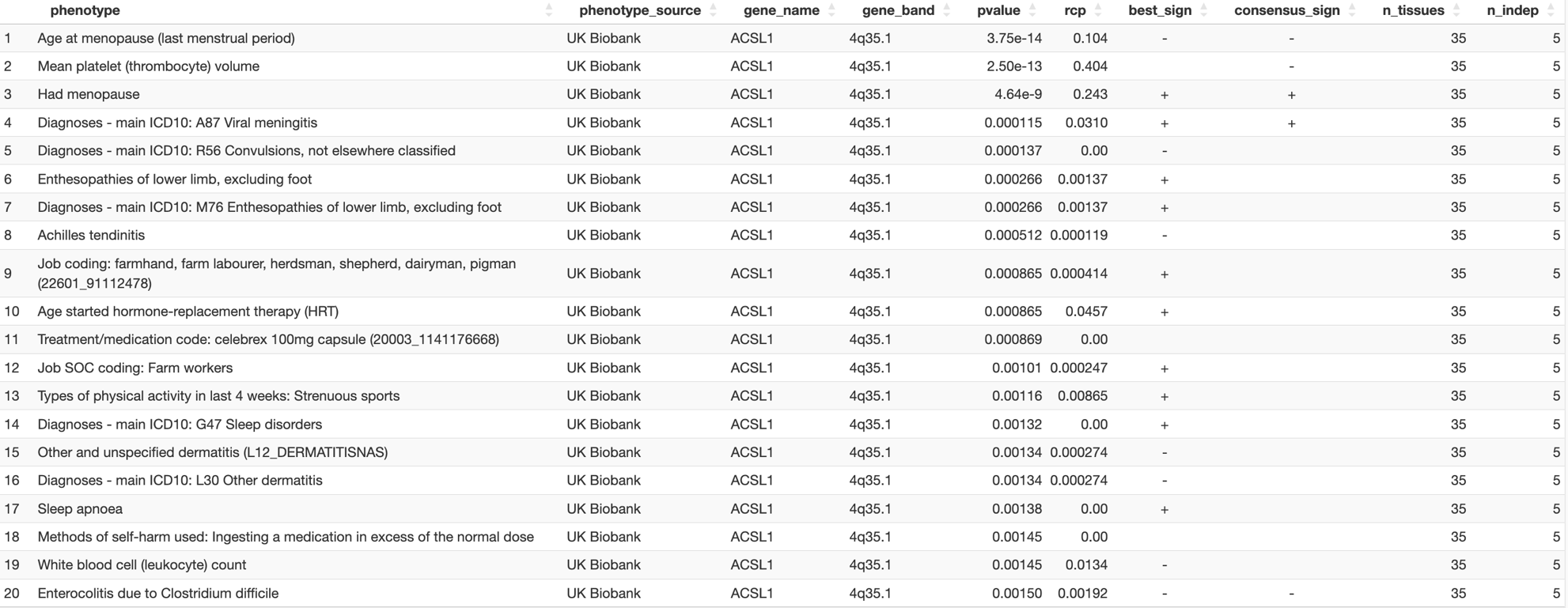


Supplementary Figure S6. Phenotypes associated with the predicted expression of *ACSL1*, as reported by PhenomeXcan. Only the top 20 associations are reported here. Columns meaning: rcp (Regional Colocalization Probability from fastENLOC/ENLOC; zero for UK Biobank means <1e-4); pvalue (p-value from S-MultiXcan); best_sign (contains the sign of effect of the most significant tissue; '+' ('-') means higher (lower) expression is associated with higher risk or higher value of phenotype); consensus_sign (contains the sign of effect of the consensus among those tissues with pvalue < 1e-4; '+' and '-' mean the same as in the best_sign column); n_tissues (number of tissues available to S-MultiXcan when computing significance for a gene); n_indep (number of independent components of variations among n_tissues)

Supplementary Table S1. Results of phenome-wide association study of *ACSL1* in GWAS Catalog.

| STRONGEST SNP  -RISK ALLELE | CHR | CHR_POS | P-VALUE | OR or BETA | 95% CI (TEXT) | DISEASE/TRAIT | Group | PUBMED  ID | DATE ADDED TO CATALOG |
| --- | --- | --- | --- | --- | --- | --- | --- | --- | --- |
| rs12503643-? | 4 | 184824934 | 6.00E-25 |  |  | Age at menopause | Menopause | 30595370 | 2/8/19 |
| rs146302237-AT | 4 | 184792442 | 3.00E-24 | 0.0349 | [0.028-0.042]  unit decrease | Hemoglobin A1c levels | Diabetes/  Glucose | 34594039 | 12/14/21 |
| rs34237618-T | 4 | 184842156 | 7.00E-20 | 0.023 | [0.018-0.028]  SD unit increase | Mean platelet volume | Blood-cell traits | 32888493 | 9/21/20 |
| rs72695645-G | 4 | 184792454 | 9.00E-20 | 0.0611 | [0.048-0.074]  unit increase | Type 2 diabetes | Diabetes/  Glucose | 32541925 | 9/14/20 |
| rs55691245-G | 4 | 184794946 | 2.00E-18 | 0.0561 | [0.044-0.069]  unit increase | Type 2 diabetes | Diabetes/  Glucose | 32541925 | 9/14/20 |
| rs116401167-C | 4 | 184794531 | 7.00E-18 | 0.0312 | [0.024-0.038]  unit decrease | Glucose levels | Diabetes/  Glucose | 34594039 | 12/14/21 |
| rs6856693-A | 4 | 184827652 | 1.00E-16 | 0.16 | [0.12-0.2]  years decrease | Menopause (age at onset) | Menopause | 26414677 | 2/21/18 |
| rs72695652-A | 4 | 184794747 | 6.00E-16 | 0.0035 | [0.0027-0.0043]  unit increase | Random glucose levels | Diabetes/  Glucose | 37679419 | 12/6/23 |
| rs72695652-A | 4 | 184794747 | 9.00E-16 | 0.00345 | [0.0026-0.0043]  unit increase | Random glucose levels | Diabetes/  Glucose | 37679419 | 12/6/23 |
| rs12503643-T | 4 | 184824934 | 5.00E-14 | 0.283 | [0.21-0.36]  unit increase | Years ovulating | Menopause | 36320039 | 2/23/23 |
| rs1996546-G | 4 | 184793135 | 9.00E-14 | 0.069 | [0.051-0.087]  unit increase | Type 2 diabetes | Diabetes/  Glucose | 35551307 | 8/19/22 |
| rs58730668-T | 4 | 184796605 | 1.00E-13 | 1.07 | [1.05-1.09] | Type 2 diabetes | Diabetes/  Glucose | 30297969 | 1/8/20 |
| rs12513029-? | 4 | 184743964 | 2.00E-13 |  |  | Neutrophil perturbation response | Blood-cell traits | 38049662 | 12/19/23 |
| rs1996546-G | 4 | 184793135 | 6.00E-13 | 1.06 | [1.05-1.08] | Type 2 diabetes | Diabetes/  Glucose | 35551307 | 8/19/22 |
| rs6552828-A | 4 | 184804262 | 2.00E-12 | 0.0044 | [0.0032-0.0056]  unit increase | Height | Others | 36224396 | 2/9/23 |
| rs735949-C | 4 | 184795078 | 2.00E-11 | 0.0711 | [0.05-0.092]  unit decrease | Type 2 diabetes | Diabetes/  Glucose | 30054458 | 1/11/19 |
| rs146302237-? | 4 | 184792442 | 2.00E-11 | 0.0096 | [0.0068-0.0124]  unit decrease | Medication use for T2D | Diabetes/  Glucose | 36653479 | 3/8/23 |
| rs60780116-T | 4 | 184787653 | 2.00E-10 | 1.09 | [1.06-1.12] | Type 2 diabetes | Diabetes/  Glucose | 28566273 | 9/22/17 |
| rs4862423-T | 4 | 184805394 | 4.00E-10 | 0.0123 | [0.0086-0.016]  unit increase | Fasting glucose | Diabetes/  Glucose | 34059833 | 6/10/21 |
| rs55881843-G | 4 | 184794936 | 2.00E-09 | 0.035 | [0.024-0.047]  unit increase | Glucose levels | Diabetes/  Glucose | 35213538 | 4/27/22 |
| rs11430387-TG | 4 | 184815490 | 5.00E-09 | 0.021 | [0.014-0.028]  unit decrease | Mean platelet volume | Blood-cell traits | 32888494 | 9/18/20 |
| rs191540449-? | 4 | 184781536 | 2.00E-08 |  |  | DNA methylation variation (age effect) | Others | 30348214 | 12/3/18 |
| rs12644905-C | 4 | 184755529 | 4.00E-06 | 1.91 | [1.45-2.52] | Fulminant type 1 diabetes | Diabetes/  Glucose | 30552108 | 3/27/19 |
| rs7660927-C | 4 | 184793802 | 5.00E-06 | 1.08 | [1.05-1.12] | Neuroblastoma or malignant cutaneous melanoma | Cancer | 31605138 | 7/9/20 |

Supplementary Table S2. Results of phenome-wide association study of *ACSL1* in Open Targets Genetics.

| RSID | P-Value | Beta or OR | CI Lower | CI Upper | Reported Trait | Group | L2G Score | PMID |
| --- | --- | --- | --- | --- | --- | --- | --- | --- |
| rs72695653 | 2.30E-33 | -0.034 | -0.039 | -0.028 | Hemoglobin A1c levels | Diabetes/Glucose | 0.78 | 34226706 |
| rs34277324 | 2.60E-28 | -0.022 | -0.026 | -0.017 | Mean platelet volume | Blood-cell traits | 0.76 | 34226706 |
| rs4862437 | 3.50E-28 | 0.020 | 0.016 | 0.024 | Mean platelet volume | Blood-cell traits | 0.31 | 34226706 |
| rs4862423 | 2.40E-26 | 0.021 | 0.017 | 0.025 | Hemoglobin A1c levels | Diabetes/Glucose | 0.82 | 34226706 |
| rs13103606 | 6.10E-26 | -0.024 | -0.028 | -0.019 | Mean platelet volume | Blood-cell traits | 0.58 | 34226706 |
| rs12503643 | 6.00E-25 |  |  |  | Age at menopause | Menopause | 0.84 | 30595370 |
| rs146302237 | 3.00E-24 | -0.035 | -0.042 | -0.028 | Hemoglobin A1c levels | Diabetes/Glucose | 0.82 | 34594039 |
| rs34237618 | 6.74E-20 | -0.023 | -0.028 | -0.018 | Mean platelet volume | Blood-cell traits | 0.72 | 32888493 |
| rs72695645 | 9.00E-20 | -0.061 | -0.074 | -0.048 | Type 2 diabetes | Diabetes/Glucose | 0.81 | 32541925 |
| rs62340877 | 1.10E-19 | -0.022 | -0.026 | -0.017 | Mean platelet volume | Blood-cell traits | 0.75 | 32888493 |
| rs72695653 | 3.60E-19 | -0.029 | -0.035 | -0.022 | Blood glucose levels | Diabetes/Glucose | 0.77 | 34226706 |
| rs55691245 | 2.00E-18 | -0.056 | -0.069 | -0.044 | Type 2 diabetes | Diabetes/Glucose | 0.81 | 32541925 |
| rs62340877 | 6.00E-18 |  |  |  | Mean platelet volume | Blood-cell traits | 0.76 | 32888493 |
| rs62340877 | 6.20E-18 | -0.022 | -0.027 | -0.017 | Mean platelet volume | Blood-cell traits | 0.72 | 32888494 |
| rs116401167 | 7.00E-18 | -0.031 | -0.038 | -0.024 | Glucose levels | Diabetes/Glucose | 0.81 | 34594039 |
| rs12503643 | 1.25E-17 | 0.183 | 0.141 | 0.224 | Age at menopause (last menstrual period) | Menopause | 0.79 |  |
| rs62340877 | 1.30E-17 | -0.023 | -0.028 | -0.017 | Plateletcrit | Blood-cell traits | 0.72 | 32888494 |
| rs35313914 | 7.79E-17 | -0.028 | -0.035 | -0.021 | Mean platelet (thrombocyte) volume | Blood-cell traits | 0.67 |  |
| rs6856693 | 1.00E-16 | 0.160 | 0.122 | 0.198 | Menopause (age at onset) | Menopause | 0.75 | 26414677 |
| rs35313914 | 1.30E-16 | -0.022 | -0.028 | -0.017 | Mean platelet volume | Blood-cell traits | 0.65 | 32888494 |
| rs58730668 | 1.00E-13 | 0.935 | 0.918 | 0.951 | Type 2 diabetes | Diabetes/Glucose | 0.83 | 30297969 |
| rs66772971 | 1.33E-13 | 0.026 | 0.019 | 0.033 | Mean platelet (thrombocyte) volume | Blood-cell traits | 0.35 |  |
| rs34277324 | 1.10E-12 | 0.017 | 0.012 | 0.022 | Mean reticulocyte volume | Blood-cell traits | 0.74 | 34226706 |
| rs145931056 | 1.90E-11 | 0.018 | 0.013 | 0.023 | Mean reticulocyte volume | Blood-cell traits | 0.75 | 32888494 |
| rs735949 | 1.95E-11 | 0.931 | 0.912 | 0.951 | Type 2 diabetes | Diabetes/Glucose | 0.75 | 30054458 |
| rs890195 | 2.00E-11 |  |  |  | Adolescent idiopathic scoliosis | Others | 0.08 | 30019117 |
| rs870825 | 2.00E-11 | -0.396 | -0.511 | -0.280 | Blood protein levels [EA, Caspase-3] | Others | 0.13 | 28240269 |
| rs34309875 | 3.37E-11 | 0.970 | 0.962 | 0.979 | Had menopause | Menopause | 0.72 |  |
| rs7665170 | 9.48E-11 | -0.001 | -0.001 | -0.001 | Platelet crit | Blood-cell traits | 0.69 |  |
| rs11721999 | 9.90E-11 | -0.007 | -0.009 | -0.005 | Sex hormone-binding globulin levels adjusted for BMI | Menopause | 0.01 | 32042192 |
| rs2720378 | 1.00E-10 |  |  |  | Kawasaki disease | Kawasaki disease | 0.03 | 33772158 |
| rs6552840 | 1.46E-10 | -0.012 | -0.016 | -0.009 | Mean platelet volume | Blood-cell traits | 0.67 | 32888493 |
| rs60780116 | 2.00E-10 | 0.917 | 0.893 | 0.942 | Type 2 diabetes | Diabetes/Glucose | 0.73 | 28566273 |
| rs4862423 | 4.00E-10 | 0.012 | 0.008 | 0.016 | Fasting glucose | Diabetes/Glucose | 0.87 | 34059833 |
| rs149150643 | 4.11E-10 | 0.880 | 0.845 | 0.916 | Hypertensive diseases | Hypertension | 0.12 |  |
| rs149150643 | 4.81E-10 | 0.880 | 0.845 | 0.916 | Hypertension | Hypertension | 0.12 |  |
| rs7672676 | 7.00E-10 |  |  |  | Adolescent idiopathic scoliosis | Others | 0.70 | 30019117 |
| rs4629495 | 1.00E-09 | 0.055 | 0.037 | 0.072 | Medication use (antithrombotic agents) | Others | 0.01 | 34594039 |
| rs34323865 | 1.00E-09 | -0.097 | -0.128 | -0.066 | Phosphatidylcholine-O_44:5_[M+H]1+/Phosphatidylcholine-P_44:4_[M+H]1+ levels | Phospholipids | 0.72 | 34503513 |
| rs745805 | 1.05E-09 | 0.919 | 0.894 | 0.944 | Type 2 diabetes, definitions combined, including avohilmo | Diabetes/Glucose | 0.76 |  |
| rs149150643 | 1.16E-09 | 0.875 | 0.839 | 0.914 | Hypertension, essential | Hypertension | 0.13 |  |
| rs4862423 | 1.83E-09 | 1.061 | 1.040 | 1.081 | Type 2 diabetes | Diabetes/Glucose | 0.81 |  |
| rs11730272 | 2.00E-09 |  |  |  | Neuritic plaques (SNP x SNP interaction) | Others | 0.02 | 32450446 |
| rs56058420 | 2.96E-09 | 0.012 | 0.008 | 0.016 | Monocyte count | Blood-cell traits | 0.05 | 32888493 |
| rs148576487 | 3.20E-09 | 0.324 | 0.217 | 0.432 | 1-palmitoyl-2-linoleoyl-gpc (16:0/18:2) levels | Phospholipids | 0.07 | 33437055 |
| rs4862423 | 3.57E-09 | 1.063 | 1.041 | 1.084 | Type 2 diabetes, strict (exclude DM1) | Diabetes/Glucose | 0.77 |  |
| rs4862423 | 3.58E-09 | 1.062 | 1.041 | 1.084 | Type 2 diabetes, definitions combined | Diabetes/Glucose | 0.77 |  |
| rs1425553 | 5.34E-09 | -0.011 | -0.015 | -0.007 | Monocyte count | Blood-cell traits | 0.03 | 32888493 |
| rs7685731 | 7.80E-09 | -0.027 | -0.036 | -0.018 | Plateletcrit | Blood-cell traits | 0.72 | 27863252 |
| rs12172978 | 9.64E-09 | 0.965 | 0.954 | 0.977 | Other exercises (eg: swimming, cycling, keep fit, bowling) \| types of physical activity in last 4 weeks | Others | 0.01 |  |
| rs4862423 | 1.68E-08 | 1.054 | 1.035 | 1.074 | Diabetes mellitus | Diabetes/Glucose | 0.76 |  |
| rs34323865 | 1.90E-08 | -0.013 | -0.018 | -0.009 | Basophil percentage of white cells | Blood-cell traits | 0.71 | 32888494 |
| rs2720378 | 2.00E-08 |  |  |  | Kawasaki disease | Kawasaki disease | 0.05 | 33106546 |
| rs12644686 | 2.44E-08 | 0.898 | 0.864 | 0.933 | Type 1 diabetes | Diabetes/Glucose | 0.01 | 34012112 |
| rs6823934 | 2.58E-08 | 0.746 | 0.673 | 0.827 | Lesion of sciatic nerve | Others | 0.41 |  |
| rs188407139 | 2.99E-08 | 0.434 | 0.280 | 0.587 | 6mm regularity index (right) | Others | 0.01 |  |
| rs2130392 | 3.00E-08 | 1.420 | 1.254 | 1.608 | Kawasaki disease | Kawasaki disease | 0.04 | 22446962 |
| rs4862423 | 3.30E-08 | 1.053 | 1.034 | 1.072 | Diabetes, varying definitions | Diabetes/Glucose | 0.76 |  |
| rs572655515 | 3.69E-08 | 1.308 | 1.189 | 1.439 | Sleep disorders (combined) | Others | 0.77 |  |
| rs116910459 | 4.00E-08 | 1.320 | 1.195 | 1.458 | Nonsyndromic cleft lip | Others | 0.02 | 31609978 |
| rs57214277 | 4.00E-08 | 1.130 | 1.082 | 1.180 | Chronic lymphocytic leukemia | Cancer | 0.06 | 28165464 |
| rs4862423 | 4.07E-08 | 1.063 | 1.040 | 1.086 | Type 2 diabetes with other specified/multiple/unspecified complications | Diabetes/Glucose | 0.43 |  |
| rs13152116 | 4.67E-08 | 0.106 | 0.068 | 0.144 | Mean reticulocyte volume | Blood-cell traits | 0.39 |  |
| rs62345895 | 4.92E-08 | 0.875 | 0.834 | 0.918 | Hypertensive diseases (excluding secondary) | Hypertension | 0.15 |  |
| rs12505298 | 4.92E-08 | -0.015 | -0.020 | -0.009 | Mean platelet (thrombocyte) volume | Blood-cell traits | 0.10 |  |

Supplementary Table S3. Results of phenome-wide association study of *ACSL1* in GWAS ATLAS.

| PMID | Year | Domain | Trait | P-value | N |
| --- | --- | --- | --- | --- | --- |
| 27864402 | 2016 | Activities | Self-rated health | 0.015706 | 111749 |
| 31427789 | 2019 | Activities | Duration of vigorous activity | 0.00279 | 210896 |
| 31427789 | 2019 | Activities | Hands-free device/speakerphone use with mobile phone in last 3 month | 0.041965 | 324453 |
| 31427789 | 2019 | Activities | Breastfed as a baby | 0.01972 | 293760 |
| 31427789 | 2019 | Activities | Type of accommodation lived in: A house or bungalow | 0.00016789 | 386168 |
| 31427789 | 2019 | Activities | Type of accommodation lived in: A flat, maisonette or apartment | 0.00074748 | 386168 |
| 31427789 | 2019 | Activities | Own or rent accommodation lived in: Own outright (by you or someone in your household) | 0.0085337 | 383032 |
| 31427789 | 2019 | Activities | Own or rent accommodation lived in: Own with a mortgage | 0.011324 | 383032 |
| 31427789 | 2019 | Activities | Gas or solid-fuel cooking/heating: A gas hob or gas cooker | 0.0065019 | 385289 |
| 31427789 | 2019 | Activities | Reason for glasses/contact lenses: For short-sightedness, i.e. only or mainly for distance viewing such as driving, cinema etc (called 'myopia') | 0.013431 | 78647 |
| 31427789 | 2019 | Activities | Treatment/medication code: omega-3/fish oil supplement | 0.026109 | 280443 |
| 31427789 | 2019 | Activities | Treatment/medication code: lisinopril | 0.020335 | 280443 |
| 28358823 | 2017 | Body Structures | Anterior cruciate ligament rupture (fixed effect model) | 0.0019869 | 99342 |
| 28358823 | 2017 | Body Structures | Anterior cruciate ligament rupture (random effect model) | 0.0022893 | 99342 |
| 31427789 | 2019 | Cardiovascular | Pulse rate (automated reading) | 0.00010474 | 361411 |
| 31427789 | 2019 | Cardiovascular | Vascular/heart problems diagnosed by doctor: High blood pressure | 0.013759 | 385699 |
| 31217584 | 2019 | Cardiovascular | Diastolic Blood Pressure | 0.015979 | 35433 |
| 30940143 | 2019 | Cardiovascular | Resting heart rate | 0.000094048 | 458969 |
| 28240269 | 2017 | Cell | TIMP3 - Metalloproteinase inhibitor 3 | 0.027157 | 1000 |
| 28240269 | 2017 | Cell | IGFBP2 - Insulin-like growth factor-binding protein 2 | 0.0078904 | 1000 |
| 28240269 | 2017 | Cell | MPO - Myeloperoxidase | 0.0053603 | 1000 |
| 28240269 | 2017 | Cell | C3 - Complement C3b, inactivated | 0.01357 | 1000 |
| 28240269 | 2017 | Cell | PLA2G2A - Phospholipase A2, membrane associated | 0.0042093 | 1000 |
| 28240269 | 2017 | Cell | PROS1 - Vitamin K-dependent protein S | 0.0033304 | 1000 |
| 28240269 | 2017 | Cell | SERPINA7 - Thyroxine-Binding Globulin | 0.032172 | 1000 |
| 28240269 | 2017 | Cell | FGF19 - Fibroblast growth factor 19 | 0.049184 | 1000 |
| 28240269 | 2017 | Cell | LTF - Lactotransferrin | 0.032585 | 1000 |
| 28240269 | 2017 | Cell | ALPL - Alkaline phosphatase, tissue-nonspecific isozyme | 0.0024961 | 1000 |
| 28240269 | 2017 | Cell | SPINT2 - Kunitz-type protease inhibitor 2 | 0.026316 | 1000 |
| 28240269 | 2017 | Cell | ARG1 - Arginase-1 | 0.030953 | 1000 |
| 28240269 | 2017 | Cell | CHEK1 - Serine/threonine-protein kinase Chk1 | 0.039077 | 1000 |
| 28240269 | 2017 | Cell | HDAC8 - Histone deacetylase 8 | 0.040803 | 1000 |
| 28240269 | 2017 | Cell | MDK - Midkine | 0.01498 | 1000 |
| 28240269 | 2017 | Cell | CYCS - Cytochrome c | 0.0062554 | 1000 |
| 28240269 | 2017 | Cell | GHR - Growth hormone receptor | 0.01297 | 1000 |
| 28240269 | 2017 | Cell | APP - Amyloid beta A4 protein | 0.030268 | 1000 |
| 28240269 | 2017 | Cell | F7 - Coagulation Factor VII | 0.041039 | 1000 |
| 28240269 | 2017 | Cell | TFPI - Tissue factor pathway inhibitor | 0.023098 | 1000 |
| 28240269 | 2017 | Cell | THBS4 - Thrombospondin-4 | 0.010953 | 1000 |
| 28240269 | 2017 | Cell | CTSV - Cathepsin L2 | 0.036931 | 1000 |
| 28240269 | 2017 | Cell | PIK3CG - Phosphatidylinositol 4,5-bisphosphate 3-kinase catalytic subunit gamma isoform | 0.0056411 | 1000 |
| 28240269 | 2017 | Cell | STAB2 - Stabilin-2 | 0.043202 | 1000 |
| 28240269 | 2017 | Cell | AGT - Angiotensinogen | 0.016711 | 1000 |
| 28240269 | 2017 | Cell | VIP - Vasoactive Intestinal Peptide | 0.034022 | 1000 |
| 28240269 | 2017 | Cell | CDH6 - Cadherin-6 | 0.02089 | 1000 |
| 28240269 | 2017 | Cell | CASP3 - Caspase-3 | 0.00042957 | 1000 |
| 28240269 | 2017 | Cell | PRSS27 - Serine protease 27 | 0.0004878 | 1000 |
| 28240269 | 2017 | Cell | CDC42BPB - Serine/threonine-protein kinase MRCK beta | 0.039255 | 1000 |
| 28240269 | 2017 | Cell | TEC - Tyrosine-protein kinase Tec | 0.04278 | 1000 |
| 28240269 | 2017 | Cell | KLK14 - Kallikrein-14 | 0.041097 | 1000 |
| 28240269 | 2017 | Cell | AIP - AH receptor-interacting protein | 0.0087823 | 1000 |
| 28240269 | 2017 | Cell | SYNCRIP - Heterogeneous nuclear ribonucleoprotein Q | 0.0009337 | 1000 |
| 28240269 | 2017 | Cell | DYRK3 - Dual specificity tyrosine-phosphorylation-regulated kinase 3 | 0.005152 | 1000 |
| 28240269 | 2017 | Cell | ENTPD3 - Ectonucleoside triphosphate diphosphohydrolase 3 | 0.024772 | 1000 |
| 28240269 | 2017 | Cell | SPHK2 - Sphingosine kinase 2 | 0.0041942 | 1000 |
| 28240269 | 2017 | Cell | NCAM1 - Neural cell adhesion molecule 1, 120 kDa isoform | 0.0056363 | 1000 |
| 28240269 | 2017 | Cell | AFM - Afamin | 0.042776 | 1000 |
| 28240269 | 2017 | Cell | SERPINA6 - Corticosteroid-binding globulin | 0.039523 | 1000 |
| 28240269 | 2017 | Cell | SHBG - Sex hormone-binding globulin | 0.0040258 | 1000 |
| 28240269 | 2017 | Cell | RPSA - 40S ribosomal protein SA | 0.017912 | 1000 |
| 28240269 | 2017 | Cell | FGFR4 - Fibroblast growth factor receptor 4 | 0.039519 | 1000 |
| 28240269 | 2017 | Cell | HRG - Histidine-rich glycoprotein | 0.034303 | 1000 |
| 28240269 | 2017 | Cell | LILRB1 - Leukocyte immunoglobulin-like receptor subfamily B member 1 | 0.01524 | 1000 |
| 28240269 | 2017 | Cell | NOTCH3 - Neurogenic locus notch homolog protein 3 | 0.029523 | 1000 |
| 28240269 | 2017 | Cell | ABL2 - Abelson tyrosine-protein kinase 2 | 0.042934 | 1000 |
| 28240269 | 2017 | Cell | CDH15 - Cadherin-15 | 0.028746 | 1000 |
| 28240269 | 2017 | Cell | CD27 - CD27 antigen | 0.012873 | 1000 |
| 30053915 | 2018 | Cell | Mumps IgG levels | 0.012429 | 921 |
| 31427789 | 2019 | Cognitive | Fluid intelligence score | 0.049711 | 125935 |
| 31427789 | 2019 | Connective Tissue | Diagnoses - secondary ICD10: M13 Other arthritis | 0.017472 | 244890 |
| 31427789 | 2019 | Dermatological | Ease of skin tanning | 2.0507E-06 | 378364 |
| 30674883 | 2019 | Dermatological | Vitiligo (late onset) | 0.0029565 | 20623 |
| 30573740 | 2018 | Dermatological | Male pattern baldness (BOLT LMM non-infinitesimal mixed model) | 0.037488 | 205327 |
| 31427789 | 2019 | Ear, Nose, Throat | Hearing difficulty/problems | 0.013482 | 370713 |
| 31427789 | 2019 | Ear, Nose, Throat | Hearing difficulty/problems with background noise | 0.04625 | 378722 |
| 27416945 | 2016 | Endocrine | Insulin sensitivity index (adjusted for age, sex. Bmi) | 0.030026 | 16753 |
| 27416945 | 2016 | Endocrine | Insulin sensitivity index (combined influence of the genotype effect adjusted for BMI and the interaction effect between the genotype and BMI on ISI) | 0.049507 | 16753 |
| 22885922 | 2012 | Endocrine | Type 2 Diabetes | 0.001924 | 69033 |
| 27398621 | 2016 | Endocrine | Type 2 Diabetes | 2.60E-08 | 44414 |
| 24934506 | 2014 | Endocrine | Skin fluorescence | 0.03979 | 1082 |
| 31427789 | 2019 | Endocrine | Diabetes (diagnosed by doctor) | 0.00079326 | 385420 |
| 31427789 | 2019 | Endocrine | Non-cancer illness code, self-reported: diabetes | 0.019864 | 289307 |
| 31427789 | 2019 | Endocrine | Diagnoses - secondary ICD10: E11 Type 2 diabetes mellitus | 0.0032263 | 244890 |
| 29358691 | 2018 | Endocrine | Type 2 Diabetes | 0.00016475 | 70127 |
| 30054458 | 2018 | Endocrine | Type 2 Diabetes | 6.20E-10 | 659256 |
| 28566273 | 2017 | Endocrine | Type 2 Diabetes | 3.14E-11 | 159208 |
| 28566273 | 2017 | Endocrine | Type 2 Diabetes (adjusted for BMI) | 2.39E-10 | 159208 |
| 30297969 | 2018 | Endocrine | Type 2 Diabetes | 1.13E-15 | 898130 |
| 30297969 | 2018 | Endocrine | Type 2 Diabetes (adjusted for BMI) | 8.60E-14 | 898130 |
| 30718926 | 2019 | Endocrine | Type 2 Diabetes | 0.0047234 | 191764 |
| 30367059 | 2018 | Endocrine | Thyroid-stimulating hormone | 0.025317 | 54288 |
| 30367059 | 2018 | Endocrine | Thyroid-stimulating hormone (female) | 0.022018 | 30236 |
| 31427789 | 2019 | Environment | Non-accidental death in close genetic family | 0.014013 | 122322 |
| 31427789 | 2019 | Environment | Illnesses of father: Diabetes | 0.00046944 | 355137 |
| 31427789 | 2019 | Environment | Illnesses of mother: High blood pressure | 0.014984 | 367939 |
| 31427789 | 2019 | Environment | Illnesses of mother: Diabetes | 0.025143 | 367939 |
| 31427789 | 2019 | Environment | Illnesses of mother: Severe depression | 0.010631 | 367939 |
| 29777097 | 2018 | Environment | Family history of Alzheimer's disease | 0.015932 | 314278 |
| 31015401 | 2019 | Environmental | Drugs used in diabetes | 0.01026 | 305913 |
| 28067908 | 2017 | Gastrointestinal | Crohn's Disease | 0.040171 | 40266 |
| 28067908 | 2017 | Gastrointestinal | Inflammatory Bowel Disease | 0.023149 | 59957 |
| 31427789 | 2019 | Gastrointestinal | Non-cancer illness code, self-reported: gastro-oesophageal reflux (gord) / gastric reflux | 0.010845 | 289307 |
| 31427789 | 2019 | Gastrointestinal | Diagnoses - secondary ICD10: K57 Diverticular disease of intestine | 0.010051 | 244890 |
| 23000144 | 2012 | Gastrointestinal | Primary biliary cirrhosis | 0.044196 | 963 |
| 30281099 | 2018 | Gastrointestinal | Infantile hypertrophic pyloric stenosis | 0.0024004 | 5833 |
| 20858683 | 2010 | Hematological | Hemoglobin A1C | 0.0040222 | 46368 |
| 25772697 | 2015 | Immunological | Lymph:%DP T | 0.025366 | 669 |
| 25772697 | 2015 | Immunological | CD4:%TM (1) | 0.023328 | 669 |
| 25772697 | 2015 | Immunological | CD4nv:%TFH (2) | 0.036672 | 669 |
| 27863252 | 2016 | Immunological | Basophil percentage of granulocytes (two-way meta) | 0.035663 | 131536 |
| 27863252 | 2016 | Immunological | Eosinophil percentage of white cells (two-way meta) | 0.033752 | 132052 |
| 27863252 | 2016 | Immunological | Eosinophil percentage of granulocytes (two-way meta) | 0.038039 | 131525 |
| 27863252 | 2016 | Immunological | Mean corpuscular hemoglobin (two-way meta) | 0.045407 | 132224 |
| 27863252 | 2016 | Immunological | Mean corpuscular volume (two-way meta) | 0.0076317 | 132353 |
| 27863252 | 2016 | Immunological | Mean platelet volume (two-way meta) | 0.00026452 | 127230 |
| 27863252 | 2016 | Immunological | Neutrophil percentage of granulocytes (two-way meta) | 0.010641 | 131660 |
| 27863252 | 2016 | Immunological | Red blood cell count (two-way meta) | 0.0068451 | 132690 |
| 27863252 | 2016 | Immunological | Basophil percentage of granulocytes (three-way meta) | 0.038321 | 170223 |
| 27863252 | 2016 | Immunological | Mean corpuscular volume (three-way meta) | 0.021186 | 172433 |
| 27863252 | 2016 | Immunological | Mean platelet volume (three-way meta) | 0.00039699 | 164454 |
| 27863252 | 2016 | Immunological | Neutrophil percentage of granulocytes (three-way meta) | 0.029607 | 170672 |
| 29403010 | 2018 | Immunological | Basophil count | 0.040947 | 62076 |
| 30053915 | 2018 | Immunological | Rubella seropositivity | 0.010986 | 1000 |
| 20018961 | 2009 | Infection | Leprosy | 0.021299 | 1220 |
| 26426971 | 2015 | Metabolic | Waist-hip ratio (male > 50 yrs, adjusted for BMI) | 0.028688 | 74324 |
| 25673412 | 2015 | Metabolic | Waist-hip ratio (male, adjusted for BMI) | 0.046411 | 93480 |
| 25673412 | 2015 | Metabolic | Waist-hip ratio (male, adjusted for BMI) | 0.046869 | 99078 |
| 26831199 | 2016 | Metabolic | Estimated glomerular filtration rate based on serum creatinine | 0.020384 | 16474 |
| 24816252 | 2014 | Metabolic | Amino acid::Alanine and aspartate metabolism::alanine | 0.01471 | 7788 |
| 24816252 | 2014 | Metabolic | Amino acid::Valine, leucine and isoleucine metabolism::levulinate (4-oxovalerate) | 0.015865 | 6982 |
| 24816252 | 2014 | Metabolic | Lipid::Carnitine metabolism::2-tetradecenoyl carnitine | 0.047888 | 6998 |
| 24816252 | 2014 | Metabolic | Lipid::Carnitine metabolism::cis-4-decenoyl carnitine | 0.005357 | 7660 |
| 24816252 | 2014 | Metabolic | Lipid::Carnitine metabolism::decanoylcarnitine | 0.0057566 | 7766 |
| 24816252 | 2014 | Metabolic | Lipid::Carnitine metabolism::hexanoylcarnitine | 0.026592 | 7786 |
| 24816252 | 2014 | Metabolic | Lipid::Carnitine metabolism::octanoylcarnitine | 0.00017681 | 7790 |
| 24816252 | 2014 | Metabolic | Lipid::Long chain fatty acid::palmitate (16:0) | 0.037832 | 7800 |
| 24816252 | 2014 | Metabolic | Lipid::Long chain fatty acid::stearate (18:0) | 0.044495 | 7803 |
| 24816252 | 2014 | Metabolic | Lipid::Lysolipid::1-arachidonoylglycerophosphoinositol* | 0.015351 | 7797 |
| 24816252 | 2014 | Metabolic | Lipid::Medium chain fatty acid::undecanoate (11:0) | 0.049821 | 7500 |
| 24816252 | 2014 | Metabolic | Lipid::Monoacylglycerol::1-linoleoylglycerol (1-monolinolein) | 0.043913 | 2797 |
| 24816252 | 2014 | Metabolic | Lipid::Monoacylglycerol::1-oleoylglycerol (1-monoolein) | 0.048156 | 5717 |
| 24816252 | 2014 | Metabolic | Nucleotide::Purine metabolism, (hypo)xanthine, inosine containing::hypoxanthine | 0.018337 | 7287 |
| 24816252 | 2014 | Metabolic | Xenobiotics::Benzoate metabolism::benzoate | 0.034703 | 7756 |
| 24816252 | 2014 | Metabolic | Xenobiotics::Chemical::glycerol 2-phosphate | 0.015472 | 5912 |
| 24816252 | 2014 | Metabolic | Xenobiotics::Xanthine metabolism::3-methylxanthine | 0.0097361 | 5396 |
| 24816252 | 2014 | Metabolic | ::::X-04494 | 0.01592 | 5007 |
| 24816252 | 2014 | Metabolic | ::::X-06246 | 0.01312 | 7358 |
| 24816252 | 2014 | Metabolic | ::::X-06307 | 0.018637 | 6774 |
| 24816252 | 2014 | Metabolic | ::::X-11327 | 0.046591 | 7671 |
| 24816252 | 2014 | Metabolic | ::::X-11437 | 0.012731 | 6782 |
| 24816252 | 2014 | Metabolic | ::::X-11497 | 0.022192 | 7481 |
| 24816252 | 2014 | Metabolic | ::::X-12013 | 0.017254 | 1948 |
| 24816252 | 2014 | Metabolic | ::::X-12230 | 0.037004 | 5649 |
| 24816252 | 2014 | Metabolic | ::::X-12544 | 0.045001 | 5893 |
| 24816252 | 2014 | Metabolic | ::::X-12719 | 0.0076832 | 1539 |
| 27005778 | 2016 | Metabolic | Alanine | 0.045019 | 24796 |
| 27005778 | 2016 | Metabolic | CH2 groups in fatty acids | 0.04439 | 19021 |
| 27005778 | 2016 | Metabolic | Double bonds in fatty acids | 0.013212 | 15728 |
| 27005778 | 2016 | Metabolic | Tyrosine | 0.042018 | 24925 |
| 27005778 | 2016 | Metabolic | VLDL diameter | 0.038113 | 19273 |
| 27005778 | 2016 | Metabolic | Concentration of chylomicrons and extremely large VLDL particles | 0.0019984 | 18960 |
| 27918534 | 2017 | Metabolic | Visceral sdipose tissue volume | 0.0017596 | 18332 |
| 27918534 | 2017 | Metabolic | Visceral sdipose tissue volume (female) | 0.0054619 | 9594 |
| 27918534 | 2017 | Metabolic | Visceral sdipose tissue volume (adjusted for BMI) | 0.018675 | 18332 |
| 27918534 | 2017 | Metabolic | Visceral sdipose tissue volume (adjusted for BMI, female) | 0.0042397 | 9594 |
| 27918534 | 2017 | Metabolic | Pericardial adipose tissue volume (male) | 0.033826 | 5842 |
| 27918534 | 2017 | Metabolic | Visceral adipose tissue sttenutation (female) | 0.033067 | 9594 |
| 27918534 | 2017 | Metabolic | Ratio of visceral-tosubcutaneous adipose tissue volume | 0.035636 | 18191 |
| 27918534 | 2017 | Metabolic | Ratio of visceral-tosubcutaneous adipose tissue volume (female) | 0.0037298 | 9823 |
| 27918534 | 2017 | Metabolic | Ratio of visceral-tosubcutaneous adipose tissue volume (adjusted for BMI, female) | 0.021061 | 9823 |
| 24699409 | 2014 | Metabolic | Corrected insulin response (adjusted for insulin sensitivity index) | 0.018016 | 4789 |
| 24699409 | 2014 | Metabolic | Disposition index | 0.0075644 | 5130 |
| 22581228 | 2012 | Metabolic | Fasting glucose main effect | 0.000018933 | 58074 |
| 22581228 | 2012 | Metabolic | Fasting glucose main effect (adjusted for BMI) | 3.6934E-06 | 58074 |
| 20081858 | 2010 | Metabolic | Fasting glucose | 0.00011372 | 46186 |
| 17658951 | 2007 | Metabolic | Body Mass Index | 0.01633 | 1410 |
| 17658951 | 2007 | Metabolic | Weight | 0.047261 | 1410 |
| 31427789 | 2019 | Metabolic | Birth weight | 0.0054831 | 219088 |
| 31427789 | 2019 | Metabolic | Impedance measures - Body fat percentage | 0.02937 | 379615 |
| 31427789 | 2019 | Metabolic | Impedance measures - Impedance of whole body | 0.035823 | 379792 |
| 31427789 | 2019 | Metabolic | Impedance measures - Impedance of arm (right) | 0.012399 | 379786 |
| 31427789 | 2019 | Metabolic | Impedance measures - Arm fat percentage (right) | 0.027167 | 379752 |
| 31427789 | 2019 | Metabolic | Impedance measures - Trunk fat percentage | 0.011235 | 379600 |
| 31427789 | 2019 | Metabolic | Diagnoses - secondary ICD10: E78 Disorders of lipoprotein metabolism and other lipidemias | 0.024604 | 244890 |
| 28892062 | 2017 | Metabolic | Body Mass Index (male) | 0.03874 | 85894 |
| 28898252 | 2017 | Metabolic | HbA1c | 0.000012367 | 159940 |
| 28898252 | 2017 | Metabolic | HbA1c | 0.000017434 | 123665 |
| 30124842 | 2018 | Metabolic | Body Mass Index | 0.0031243 | 681275 |
| 28757204 | 2017 | Metabolic | 25-Hydroxyvitamin D level | 0.014244 | 42274 |
| 27920155 | 2017 | Metabolic | Estimated glomerular filtration rate based on serum creatinine (non-diabetic) | 0.042341 | 94677 |
| 27920155 | 2017 | Metabolic | Estimated glomerular filtration rate based on serum creatinine | 0.0094658 | 111666 |
| 30239722 | 2018 | Metabolic | Body Mass Index | 0.046262 | 806834 |
| 29403010 | 2018 | Metabolic | Blood sugar | 0.0051525 | 93146 |
| 29403010 | 2018 | Metabolic | Prothrombin time | 0.044831 | 58110 |
| 31217584 | 2019 | Metabolic | Fasting glucose | 8.29E-08 | 23911 |
| 31217584 | 2019 | Metabolic | Body Mass Index | 0.045276 | 49335 |
| 30664634 | 2019 | Metabolic | Trunk-trunk fat ratio (female) | 0.0024471 | 195068 |
| 31427789 | 2019 | Mortality | Number of self-reported non-cancer illnesses | 0.0032433 | 386581 |
| 28924153 | 2017 | Neoplasms | 11q deletion neuroblastoma | 0.027825 | 5222 |
| 30323354 | 2018 | Neoplasms | BRCA1/2-negative breast cancer | 0.011055 | 7448 |
| 28604731 | 2017 | Neurological | Insomnia (male) | 0.00084945 | 53639 |
| 16252231 | 2005 | Neurological | Parkinson disease of sibling pairs (tier 1) | 0.0171 | 886 |
| 27992416 | 2017 | Neurological | Insomnia | 0.016035 | 59128 |
| 27992416 | 2017 | Neurological | Insomnia (male) | 0.0058473 | 28683 |
| 27694991 | 2016 | Neurological | Intracranial Volume | 0.017539 | 26577 |
| 31427789 | 2019 | Neurological | Pain type(s) experienced in last month: Headache | 0.011137 | 385698 |
| 31427789 | 2019 | Neurological | Non-cancer illness code, self-reported: migraine | 0.038672 | 289307 |
| 29777097 | 2018 | Neurological | Proxy and clinically diagnosed Alzheimer's disease | 0.013508 | 388324 |
| 30617256 | 2018 | Neurological | Proxy and clinically diagnosed Alzheimer's disease | 0.0011117 | 455258 |
| 31676860 | 2019 | Neurological | Left lateral orbitofrontal | 0.044321 | 19629 |
| 31676860 | 2019 | Neurological | Left posterior cingulate | 0.0036099 | 19629 |
| 31676860 | 2019 | Neurological | Right inferior parietal | 0.024421 | 19629 |
| 31676860 | 2019 | Neurological | Right parahippocampal | 0.029294 | 19629 |
| 31676860 | 2019 | Neurological | Left lateral orbitofrontal | 0.020012 | 21821 |
| 31676860 | 2019 | Neurological | Left parahippocampal | 0.038423 | 21821 |
| 31676860 | 2019 | Neurological | Left posterior cingulate | 0.0079468 | 21821 |
| 31676860 | 2019 | Neurological | Right parahippocampal | 0.0040247 | 21821 |
| 31676860 | 2019 | Neurological | Left basal forebrain | 0.036108 | 21821 |
| BioRxiv: https://doi.org/10.1101/288568 | 2019 | Neurological | Posterior limb of internal capsule fractional anisotropy | 0.0406 | 17706 |
| BioRxiv: https://doi.org/10.1101/288585 | 2019 | Neurological | Fornix (column and body of fornix) axial diusivities | 0.036371 | 17706 |
| BioRxiv: https://doi.org/10.1101/288590 | 2019 | Neurological | Posterior limb of internal capsule axial diusivities | 0.027126 | 17706 |
| BioRxiv: https://doi.org/10.1101/288598 | 2019 | Neurological | Uncinate fasciculus axial diusivities | 0.027435 | 17706 |
| BioRxiv: https://doi.org/10.1101/288626 | 2019 | Neurological | Cingulum (hippocampus) mode of anisotropy | 0.021116 | 17706 |
| BioRxiv: https://doi.org/10.1101/288634 | 2019 | Neurological | Posterior limb of internal capsule mode of anisotropy | 0.0050565 | 17706 |
| 31427789 | 2019 | Nutritional | Pork intake | 0.039672 | 384328 |
| 31427789 | 2019 | Nutritional | Cereal type: Oat cereal (e.g. Ready Brek, porridge) | 0.046771 | 319477 |
| 28073927 | 2017 | Ophthalmological | Vertical cup-disc ratio | 0.0039545 | 8373 |
| 28073927 | 2017 | Ophthalmological | Disc area | 0.039501 | 7307 |
| 19488044 | 2009 | Psychiatric | Bipolar disorder | 0.0087013 | 1033 |
| 22952603 | 2012 | Psychiatric | Baseline positive affect factor score | 0.036038 | 381 |
| 31427789 | 2019 | Psychiatric | Nap during day | 0.010886 | 386124 |
| 31427789 | 2019 | Psychiatric | Worrier / anxious feelings | 0.041644 | 376411 |
| 31427789 | 2019 | Psychiatric | Worry too long after embarrassment | 0.0071587 | 370660 |
| 31427789 | 2019 | Psychiatric | Why stopped smoking: Health precaution | 0.025553 | 94509 |
| 31427789 | 2019 | Psychiatric | Depression - Age at first episode of depression | 0.029211 | 65776 |
| 31427789 | 2019 | Psychiatric | Depression - Recent trouble concentrating on things | 0.0095981 | 126633 |
| 31427789 | 2019 | Psychiatric | Depression - Weight change during worst episode of depression | 0.012252 | 57130 |
| 29500382 | 2018 | Psychiatric | Worry too long after embarrassment (WORR-EMB) | 0.012579 | 261094 |
| 26414677 | 2015 | Reproduction | Age at menopause | 0.000084319 | 69360 |
| 31427789 | 2019 | Reproduction | Had menopause (female) | 7.7253E-06 | 175519 |
| 31427789 | 2019 | Reproduction | Age started oral contraceptive pill (female) | 0.044594 | 165121 |
| 31427789 | 2019 | Reproduction | Age when last used oral contraceptive pill (female) | 0.039565 | 149933 |
| 31427789 | 2019 | Reproduction | Ever used hormone-replacement therapy (HRT) (female) | 0.0021719 | 208012 |
| 31427789 | 2019 | Reproduction | Age at menopause (last menstrual period) (female) | 1.07E-08 | 119160 |
| 29855537 | 2018 | Reproduction | Dysmenorrhea pain severity | 0.00023677 | 11348 |
| 29855537 | 2018 | Reproduction | Dysmenorrhea (quality of life impact) | 0.0029456 | 11348 |
| 29855537 | 2018 | Reproduction | Menstrual pain medicine use | 0.040101 | 11348 |
| 30804560 | 2019 | Respiratory | FVC | 0.0090146 | 400102 |
| 30804560 | 2019 | Respiratory | FVC | 0.010097 | 321047 |
| 26367794 | 2015 | Skeletal | Lumbar Spine BMD | 0.0023464 | 32965 |
| 18204098 | 2008 | Skeletal | Systemic Lupus Erythematosus | 0.016626 | 3094 |
| 22763110 | 2012 | Skeletal | Osteoarthritis of hip and/or knee | 0.0076087 | 18419 |
| 28869591 | 2017 | Skeletal | Estimated BMD | 0.0001721 | 142487 |
| 29559693 | 2018 | Skeletal | Osteoarthritis (self reported) | 0.029635 | 63556 |
| 30124842 | 2018 | Skeletal | Height | 0.00030436 | 693529 |
| 30048462 | 2018 | Skeletal | Heel bone mineral density | 3.47E-08 | 394929 |
| 30598549 | 2018 | Skeletal | Estimated bone mineral density from heel ultrasounds | 6.38E-08 | 426824 |
| 31427789 | 2019 | Social Interactions | Number of full sisters | 0.0088102 | 380122 |
| 31427789 | 2019 | Social Interactions | Social support - Leisure/social activities: Adult education class | 0.02417 | 385280 |

Supplementary Table S4. eQTLs or trait-associated *ACSL1* genetic variants with positive selection signals

| POP | rsID | iHS | nSL | PBS | GTEx | eQTLGen | GWAS Catalog | Open Targets Genetics |
| --- | --- | --- | --- | --- | --- | --- | --- | --- |
| AFR | rs12513029 | NS | NS | 0.34 | NS | NS | Neutrophil perturbation response; | NS |
| AFR | rs60241287 | NS | -1.909 | NS | NS | Blood | NS | NS |
| AFR | rs6855063 | NS | NS | 0.436 | NS | Blood | NS | NS |
| AFR | rs4862419 | NS | NS | 0.437 | NS | Blood | NS | NS |
| AFR | rs7660927 | NS | NS | 0.348 | NS | NS | Neuroblastoma or malignant cutaneous melanoma; | NS |
| AFR | rs7686479 | NS | -2.044 | NS | NS | Blood | NS | NS |
| AFR | rs13127985 | NS | -2.221 | NS | Whole Blood; | Blood | NS | NS |
| AFR | rs9997745 | NS | NS | 0.596 | Testis; | NS | NS | NS |
| AFR | rs56302210 | NS | NS | 0.625 | Testis; | NS | NS | NS |
| AFR | rs147169415 | NS | 1.875 | 0.325 | Cells - Cultured fibroblasts; | NS | NS | NS |
| AFR | rs6833829 | NS | NS | 0.383 | Testis; | NS | NS | NS |
| AFR | rs6857027 | NS | NS | 0.383 | Testis; | NS | NS | NS |
| AFR | rs6857505 | NS | NS | 0.383 | Testis; | NS | NS | NS |
| AFR | rs28526229 | NS | NS | 0.383 | Testis; | NS | NS | NS |
| AFR | rs28709399 | NS | NS | 0.383 | Testis; | NS | NS | NS |
| AFR | rs28654441 | NS | 1.972 | NS | Cells - Cultured fibroblasts; | NS | NS | NS |
| AFR | rs10002300 | NS | 1.968 | 0.321 | Cells - Cultured fibroblasts; | NS | NS | NS |
| AFR | rs10005356 | -1.67 | NS | NS | Testis; | NS | NS | NS |
| AFR | rs6828525 | -1.805 | NS | NS | Testis; | NS | NS | NS |
| AFR | rs6828892 | -1.805 | NS | NS | Testis; | NS | NS | NS |
| AFR | rs6829342 | -1.806 | NS | NS | Testis; | NS | NS | NS |
| AFR | rs6829776 | -1.806 | NS | NS | Testis; | NS | NS | NS |
| AFR | rs6830101 | -1.806 | NS | NS | Testis; | NS | NS | NS |
| AFR | rs6830111 | NS | 1.722 | 0.312 | Cells - Cultured fibroblasts; | NS | NS | NS |
| AFR | rs6830347 | -1.762 | NS | NS | Testis; | NS | NS | NS |
| AFR | rs6827693 | NS | NS | 0.505 | Testis; | NS | NS | NS |
| AFR | rs7658926 | NS | 1.764 | 0.312 | Cells - Cultured fibroblasts; | NS | NS | NS |
| AFR | rs6818492 | NS | NS | 0.506 | Testis; | NS | NS | NS |
| AFR | rs28488461 | NS | NS | 0.505 | Testis; | NS | NS | NS |
| AFR | rs34829317 | NS | -2.056 | NS | Testis; | NS | NS | NS |
| AFR | rs12512699 | NS | -1.76 | NS | Testis; | Blood | NS | NS |
| AFR | rs7665170 | 1.72 | NS | NS | NS | NS | NS | Platelet crit; |
| EUR | rs6854996 | NS | NS | 0.182 | NS | Blood | NS | NS |
| EUR | rs8086 | NS | NS | 0.272 | NS | Blood | NS | NS |
| EUR | rs34416324 | NS | NS | 0.179 | NS | Blood | NS | NS |
| EUR | rs1878873 | NS | NS | 0.282 | NS | Blood | NS | NS |
| EUR | rs4861631 | NS | NS | 0.277 | NS | Blood | NS | NS |
| EUR | rs2292898 | NS | -2.034 | 0.262 | NS | Blood | NS | NS |
| EUR | rs3792314 | NS | NS | 0.262 | NS | Blood | NS | NS |
| EUR | rs4862415 | NS | NS | 0.258 | NS | Blood | NS | NS |
| EUR | rs4861632 | NS | NS | 0.257 | NS | Blood | NS | NS |
| EUR | rs7691972 | NS | NS | 0.255 | NS | Blood | NS | NS |
| EUR | rs3792311 | NS | NS | 0.255 | NS | Blood | NS | NS |
| EUR | rs1803898 | NS | NS | 0.284 | NS | Blood | NS | NS |
| EUR | rs7680850 | NS | NS | 0.279 | NS | Blood | NS | NS |
| EUR | rs7659090 | NS | NS | 0.283 | NS | Blood | NS | NS |
| EUR | rs7659849 | NS | NS | 0.245 | NS | Blood | NS | NS |
| EUR | rs13137179 | NS | NS | 0.25 | NS | Blood | NS | NS |
| EUR | rs6852479 | NS | NS | 0.256 | NS | Blood | NS | NS |
| EUR | rs13144160 | NS | NS | 0.276 | Whole Blood; | Blood | NS | NS |
| EUR | rs13127985 | NS | NS | 0.262 | Whole Blood; | Blood | NS | NS |
| EUR | rs13105565 | NS | NS | 0.286 | Whole Blood; | Blood | NS | NS |
| EUR | rs13112568 | NS | NS | 0.281 | Whole Blood; | Blood | NS | NS |
| EUR | rs13120078 | NS | NS | 0.318 | Whole Blood; | Blood | NS | NS |
| EUR | rs35340636 | NS | NS | 0.28 | Whole Blood; | Blood | NS | NS |
| EUR | rs11726327 | NS | NS | 0.163 | NS | Blood | NS | NS |
| EUR | rs34202162 | NS | 1.581 | NS | NS | Blood | NS | NS |
| EUR | rs7676928 | NS | 1.615 | NS | NS | Blood | NS | NS |
| EUR | rs2139178 | -1.732 | NS | NS | Testis; | Blood | NS | NS |
| EUR | rs11940804 | -1.732 | NS | NS | Testis; | Blood | NS | NS |
| EUR | rs28582376 | -1.754 | NS | NS | Testis; | Blood | NS | NS |
| EUR | rs12648071 | -1.754 | NS | NS | NS | Blood | NS | NS |
| EUR | rs28579779 | -1.758 | NS | NS | Testis; | Blood | NS | NS |
| EUR | rs10002197 | -1.795 | NS | NS | Testis; | Blood | NS | NS |
| EUR | rs35681997 | NS | -1.839 | NS | Testis; | NS | NS | NS |
| EUR | rs34829317 | NS | -2.254 | NS | Testis; | NS | NS | NS |
| EUR | rs12512699 | NS | -2.341 | NS | Testis; | Blood | NS | NS |
| EUR | rs62338246 | NS | NS | 0.173 | Testis; | NS | NS | NS |
| EUR | rs34653881 | NS | NS | 0.192 | Testis; | Blood | NS | NS |
| EUR | rs66739414 | NS | NS | 0.213 | Testis; | NS | NS | NS |
| EUR | rs34391965 | NS | NS | 0.186 | Testis; | NS | NS | NS |
| EUR | rs59674005 | NS | NS | 0.233 | Testis; | Blood | NS | NS |
| EUR | rs13111368 | NS | NS | 0.184 | Testis; | Blood | NS | NS |
| EUR | rs13111404 | NS | NS | 0.186 | Testis; | Blood | NS | NS |
| EUR | rs13139032 | NS | NS | 0.197 | Testis; | Blood | NS | NS |
| EUR | rs36073531 | NS | NS | 0.187 | Testis; | Blood | NS | NS |
| EUR | rs34552491 | NS | NS | 0.187 | Testis; | Blood | NS | NS |
| EUR | rs35943601 | NS | NS | 0.22 | Testis; | Blood | NS | NS |
| EUR | rs35594363 | NS | NS | 0.22 | Testis; | Blood | NS | NS |
| EUR | rs145931056 | NS | NS | 0.205 | Testis; | NS | NS | Mean reticulocyte volume; |
| EUR | rs7685731 | NS | NS | 0.179 | NS | NS | NS | Plateletcrit; |
| EUR | rs34277324 | NS | NS | 0.186 | NS | NS | NS | Mean platelet volume; Mean reticulocyte volume; |
| EUR | rs62340877 | NS | NS | 0.186 | NS | NS | NS | Mean platelet volume; Plateletcrit; |
| EAS | rs12513029 | NS | NS | 0.26 | NS | NS | Neutrophil perturbation response; | NS |
| EAS | rs2171838 | NS | NS | 0.278 | NS | Blood | NS | NS |
| EAS | rs6855063 | NS | NS | 0.279 | NS | Blood | NS | NS |
| EAS | rs4862419 | NS | NS | 0.279 | NS | Blood | NS | NS |
| EAS | rs9997745 | NS | NS | 0.318 | Testis; | NS | NS | NS |
| EAS | rs1554336 | NS | NS | 0.311 | Brain - Cerebellum; | Blood | NS | NS |
| EAS | rs12504478 | NS | NS | 0.285 | Brain - Cerebellum; | NS | NS | NS |
| EAS | rs112231838 | NS | NS | 0.27 | Brain - Cerebellum; | Blood | NS | NS |
| EAS | rs56302210 | NS | NS | 0.383 | Testis; | NS | NS | NS |
| EAS | rs74335426 | NS | NS | 0.253 | Brain - Cerebellum; | NS | NS | NS |
| EAS | rs55713639 | NS | NS | 0.229 | Testis; | NS | NS | NS |
| EAS | rs56408923 | NS | NS | 0.226 | Brain - Cerebellum; | NS | NS | NS |
| EAS | rs55988439 | NS | NS | 0.24 | Brain - Cerebellum; | NS | NS | NS |
| EAS | rs6833829 | NS | NS | 0.337 | Testis; | NS | NS | NS |
| EAS | rs56226652 | NS | NS | 0.24 | Brain - Cerebellum; | NS | NS | NS |
| EAS | rs6857027 | NS | NS | 0.337 | Testis; | NS | NS | NS |
| EAS | rs6857505 | NS | NS | 0.337 | Testis; | NS | NS | NS |
| EAS | rs28526229 | NS | NS | 0.337 | Testis; | NS | NS | NS |
| EAS | rs28709399 | NS | NS | 0.337 | Testis; | NS | NS | NS |
| EAS | rs140702092 | NS | NS | 0.23 | Testis; | NS | NS | NS |
| EAS | rs62338229 | NS | NS | 0.235 | Testis; | NS | NS | NS |
| EAS | rs4862437 | -1.994 | NS | NS | NS | NS | NS | Mean platelet volume; |
| EAS | rs7665170 | 1.685 | NS | NS | NS | NS | NS | Platelet crit; |
| EAS | rs7672676 | NS | NS | 0.327 | NS | NS | NS | Adolescent idiopathic scoliosis; |
| SAS | rs2171838 | NS | NS | 0.081 | NS | Blood | NS | NS |
| SAS | rs56002880 | NS | 1.562 | NS | NS | Blood | NS | NS |
| SAS | rs7660927 | NS | -1.786 | NS | NS | NS | Neuroblastoma or malignant cutaneous melanoma; | NS |
| SAS | rs4862423 | NS | NS | 0.083 | NS | NS | Fasting glucose; | Hemoglobin A1c levels; Fasting glucose; Type 2 diabetes; "Type 2 diabetes, strict (exclude DM1)"; "Type 2 diabetes, definitions combined"; Diabetes mellitus; "Diabetes, varying definitions"; Type 2 diabetes with other specified/multiple/unspecified complications; |
| SAS | rs34202162 | NS | 1.523 | NS | NS | Blood | NS | NS |
| SAS | rs62338221 | NS | -1.983 | NS | Testis; | NS | NS | NS |
| SAS | rs9997745 | NS | NS | 0.129 | Testis; | NS | NS | NS |
| SAS | rs1554336 | NS | NS | 0.078 | Brain - Cerebellum; | Blood | NS | NS |
| SAS | rs2139178 | NS | -1.752 | NS | Testis; | Blood | NS | NS |
| SAS | rs56302210 | NS | NS | 0.176 | Testis; | NS | NS | NS |
| SAS | rs55713639 | NS | NS | 0.075 | Testis; | NS | NS | NS |
| SAS | rs6833829 | NS | NS | 0.146 | Testis; | NS | NS | NS |
| SAS | rs6857027 | NS | NS | 0.146 | Testis; | NS | NS | NS |
| SAS | rs6857505 | NS | NS | 0.146 | Testis; | NS | NS | NS |
| SAS | rs28526229 | NS | NS | 0.146 | Testis; | NS | NS | NS |
| SAS | rs28709399 | NS | NS | 0.146 | Testis; | NS | NS | NS |
| SAS | rs28582376 | -1.86 | -1.924 | NS | Testis; | Blood | NS | NS |
| SAS | rs12648071 | -1.86 | -1.923 | NS | NS | Blood | NS | NS |
| SAS | rs28579779 | -1.86 | -1.924 | NS | Testis; | Blood | NS | NS |
| SAS | rs140702092 | NS | NS | 0.074 | Testis; | NS | NS | NS |
| SAS | rs10002197 | -1.865 | -1.934 | NS | Testis; | Blood | NS | NS |
| SAS | rs35681997 | NS | -1.808 | NS | Testis; | NS | NS | NS |
| SAS | rs12512699 | -1.735 | -2.24 | NS | Testis; | Blood | NS | NS |
| SAS | rs4862437 | -1.882 | NS | NS | NS | NS | NS | Mean platelet volume; |
| SAS | rs6856034 | 1.995 | NS | NS | Testis; | NS | NS | NS |
| SAS | rs7665170 | 2.432 | 1.576 | 0.092 | NS | NS | NS | Platelet crit; |

Note: For the selection-related columns (i.e., iHS, nSL, and PBS), values are reported if they are in the top 5% or bottom 5% of the genome-wide distributions. For the eQTLs-related columns (i.e., GTEx and eQTLGen), the tissue names are reported if there are significant eQTL signals. For the GWAS-related columns (i.e., GWAS Catalog and Open Targets Genetics), the associated traits are reported. NS indicates “not significant” or “not reported”.
